# First total synthesis of chromanone A, preparation of related compounds and evaluation of their antifungal activity against *Candida albicans*, a biofilm forming agent†

**DOI:** 10.1039/d1ra02553h

**Published:** 2021-06-01

**Authors:** Iván Cortés, Estefanía Cordisco, Teodoro S. Kaufman, Maximiliano A. Sortino, Laura A. Svetaz, Andrea B. J. Bracca

**Affiliations:** Instituto de Química Rosario (IQUIR, CONICET-UNR), Facultad de Ciencias Bioquímicas y Farmacéuticas, Universidad Nacional de Rosario Suipacha 531 S2002LRK Rosario Argentina kaufman@iquir-conicet.gov.ar bracca@iquir-conicet.gov.ar; Area Farmacognosia, Facultad de Ciencias Bioquímicas y Farmacéuticas, Universidad Nacional de Rosario Suipacha 531 S2002LRK Rosario Argentina lsvetaz@fbioyf.unr.edu.ar

## Abstract

A straightforward and convenient approach for the first total syntheses of chromanone A and a related 7-OMe substituted natural product is reported. These unique C-3 substituted 2-hydroxymethyl chromones were recently isolated as fungal metabolites. Chromanone A was synthesized in 25.3% overall yield from the readily available pyrocatechol, whereas the second natural product was prepared in 39.7% global yield. A small library of chromones, including both natural products and some of their synthetic heterocyclic precursors, was evaluated against *Candida albicans* ATCC 10231, a biofilm forming agent. It was found that 8-methoxy-3-methyl-4-oxo-4*H*-chromene-2-carbaldehyde, a partially oxidized form of chromanone A, exhibited a minimum inhibitory concentration of 7.8 μg mL^−1^ and significantly inhibited the yeast's virulence factors, including the adherence to buccal epithelial cells and the secretion of phospholipases, as well as the formation of germ tubes and the generation of the hyphal pseudomycelium. In addition, despite the heterocycle exhibiting non-significant inhibition of the formation of the *Candida* biofilm, it completely inhibited the growth of *C. albicans* in preformed biofilms at 62.5 μg mL^−1^.

## Abstract 2

Chromanone A and its 7-OMe analogue were totally synthesized. 2-Formyl chromanone A derivative inhibited *C. albicans* and its virulence factors.

## Graphical abstract

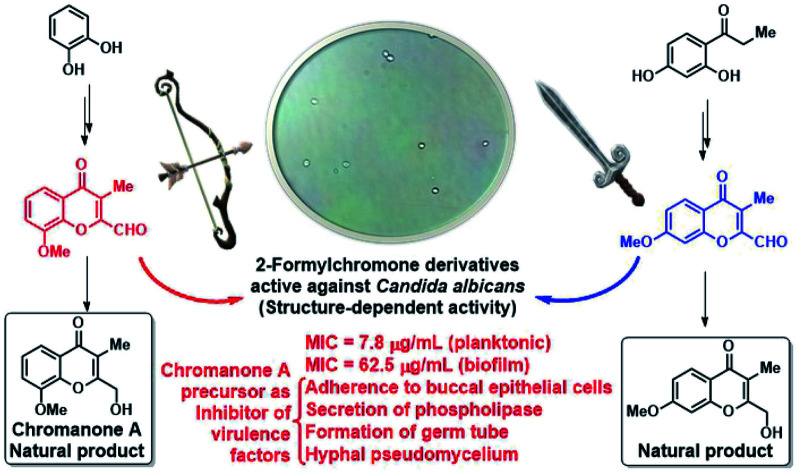

## 1. Introduction

Biofilms are structured microbial communities, embedded in a self-produced polymeric matrix of protein and polysaccharides. They are frequently found on interfaces or attached to surfaces, and are the scenario of complex interactions. These enduring structures are elusive to the defense system of the host, reluctant to undergo phagocytosis, and offer the producing microorganism a markedly increased resistance to antibiotics and chemical disinfectants.^1*a*^ Therefore, biofilms are an important form of microbial resistance and transmission of chronic infections.^1*b*^

The biofilms are ubiquitous, and their presence on surfaces such as biomedical implants, may put at risk complex surgical interventions and ultimately compromise the life of the patient. In this context, there is a need for antimicrobial agents capable of attacking microorganisms within the biofilms. Hence, the search for new compounds endowed with this property is currently relevant.

The chromone skeleton is a “privileged structure”,^2*a*^ meaning a promising motif for drug development. Not surprisingly, substituted chromones are known to display a variety of useful biological properties, including antifungal activity.^2*b*,*c*^

**Fig. 1:**
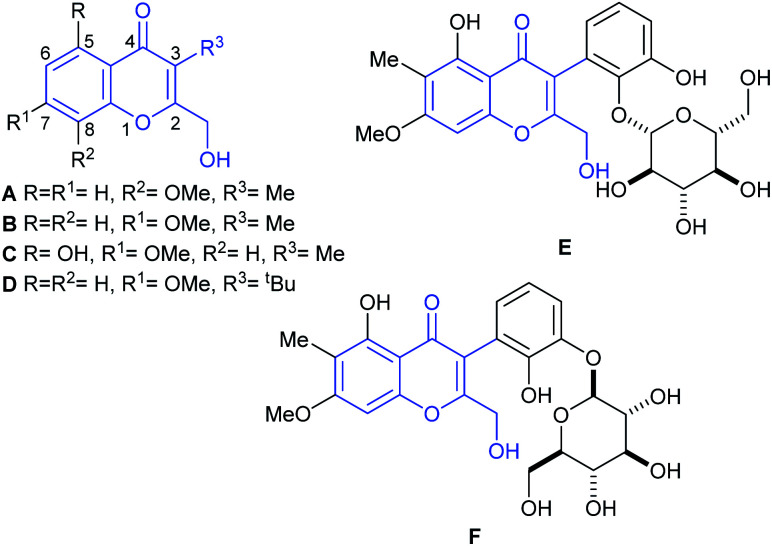
Chromanone A (A) and related naturally occurring 3-substituted-2-hydroxymethyl chromone derivatives.

In 2009, Gamal-Eldeen and coworkers reported the isolation of chromanone A (A, Fig. 1),^3^ from an algicolous marine *Penicillium* species, cultivated on a solid biomalt medium. In turn, this fungus was isolated as an endophyte of the Egyptian green alga *Ulva* sp. The natural product inhibits the activity of CYP1A at a level of 4 μg mL^−1^, being a potential cancer chemopreventive agent.^3^

The structure of the natural product was assigned based on an analysis of 1D and 2D (HSQC, HMBC) NMR spectra. Being a member of the very small family of the naturally occurring 2-hydroxymethylchromones which bear a C-3 functionalization, chromanone A is a structurally unique chromone. Its special structural characteristics are shared only by a handful of natural products, such as its isomer B, recently isolated from *Rhinocladiella* sp., a fungus obtained from the marine sponge *Ircinia oros*.^4*a*^ These features are also found in heterocycles C^4*b*^ and D,^4*c*^ as well as in boeravinone Q (E)^5*a*^ and its congener mirabijalone C (F).^5*b*^

Our work is focused on the synthesis of structurally unique heterocyclic natural products,^6^ as well as in their evaluation.^7*a*,*b*^ Further, among chromone derivatives, we have developed the total synthesis of the structure assigned to aspergillitine,^7*c*^ a 2,3-dimethyl chromone derivative isolated from a marine *Aspergillus* species.

In pursuit of these interests, here we report an efficient approach to the first total syntheses of chromanone A (A) and of its isomer, the related natural product B. The results of the evaluation of both natural products and their heterocyclic synthetic intermediates, as antifungal agents against the biofilm forming yeast *Candida albicans* ATCC 12031, are also discussed.

## 2. Results and discussion

### 2.1 Chemistry

**Scheme 1:**
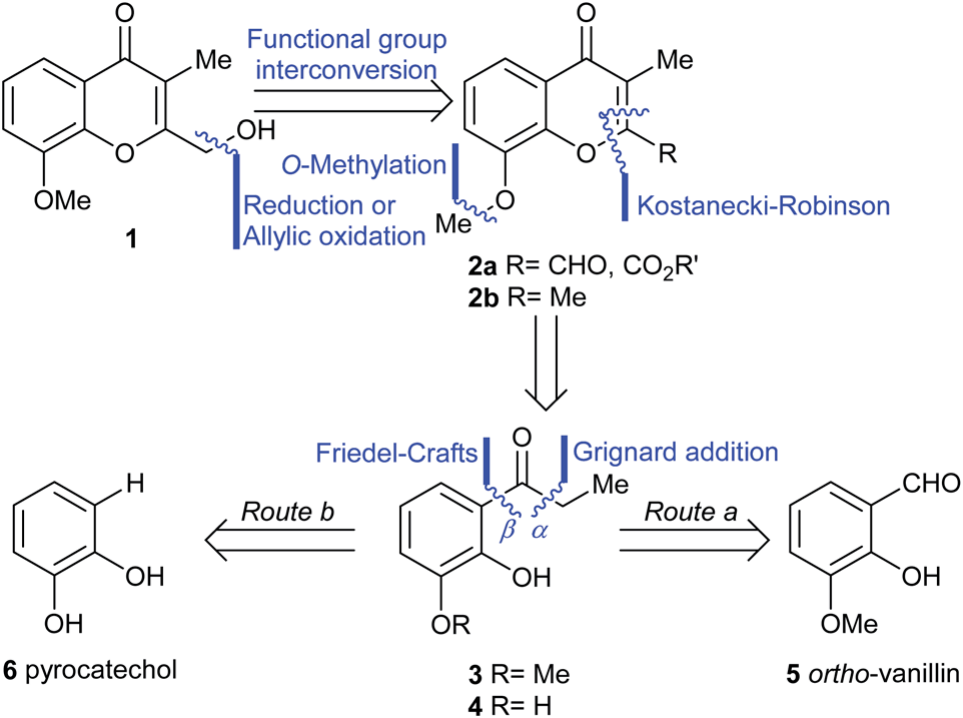
Retrosynthetic analyses of chromanone A (1).

The initial step toward chromanone A as the first target (1), was a retrosynthetic analysis of the product (Scheme 1). Initially, focus was made on the oxygen bearing functionality of the 2-hydroxymethyl feature; a functional group interconversion was proposed, conjecturing that the proper oxidation stage of the C-2 substituent could be set either through reduction (aldehyde, ester, 2a) or by means of a selective oxidation.^7*a*^ The latter possibility revealed the 2,3-dimethylchromone 2b as a suitable precursor.

Then, two different alternatives were considered. In one of them (Route a), the heterocyclic ring of 2a,b was disassembled as shown, to unveil a propiophenone derivative (3), which was further submitted to a C–O disconnection on the aliphatic side of the carbonyl moiety (bond α), to uncover *ortho*-vanillin (5) as a suitable starting material.

In the second case (Route b), an additional methyl ether C–O disconnection was considered on the chromone 2, which suggested the catechol derivative 4 as the most appropriate synthetic intermediate. In turn, a C–O disconnection on the aromatic side of the carbonyl motif of propiophenone 4 (bond β) set aside the three-carbon side chain and determined that the logical starting material for this approach was the commercially available pyrocatechol (6).

**Scheme 2:**
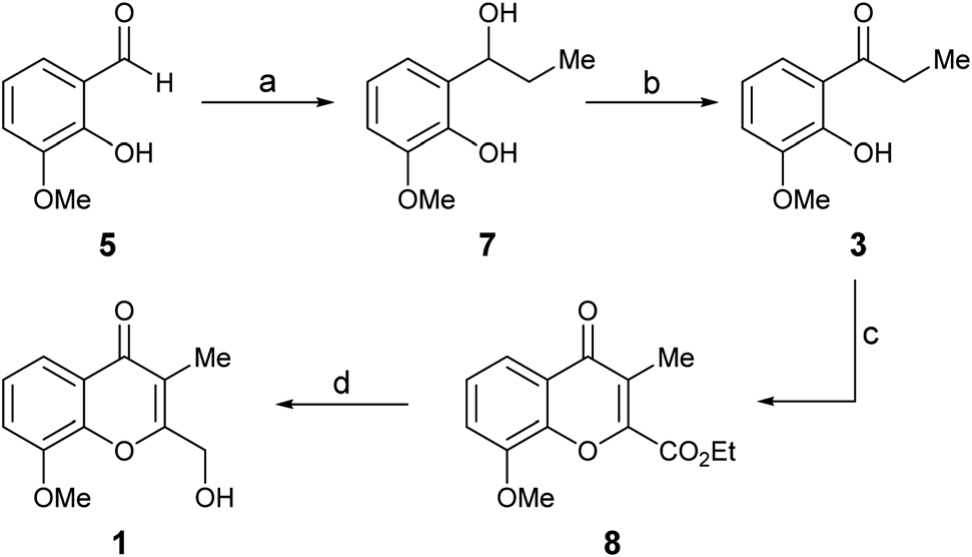
Reagents and conditions (a) CH_3_CH_2_I, Mg^0^, Et_2_O, RT (97%); (b) IBX, EtOAc, RT, 1 h (81%); (c) EtO_2_C–COCl, Et_3_N, CH_2_Cl_2_, MW (100 °C), 1 h (42%); (d) NaBH_4_, CaCl_2_, EtOH, 0 °C, 24 h (41%).

For simplicity, the Route a was explored first. Thus, *ortho*-vanillin (5) was subjected to a 1,2-addition to the carbonyl with excess ethyl Grignard reagent (Scheme 2), freshly prepared from iodoethane and activated magnesium. The reaction was executed at room temperature (RT), giving alcohol 7 in 97% yield.^8^

Subsequently, 7 was submitted to a selective oxidation of its secondary alcohol moiety. Different conditions [PDC, CH_2_Cl_2_; H_2_N–NH_2_·*x*H_2_O, DMSO/I_2_, MeCN/H_2_O (5 : 1, v/v); DMSO/Ac_2_O (2.3 equiv.); IBX (1.5 equiv.), EtOAc]^9^ were tested; however, the reaction proved most successful with IBX in EtOAc, furnishing ketone 3 in up to 81% yield in just one hour at room temperature.^10^ Unfortunately, this transformation proved to lack robustness, being highly dependent on the experimental conditions, including the source of the oxidizing reagent. This characteristic frequently resulted in an exacerbated concomitant oxidation of the *ortho*-phenol, ultimately causing tarry materials and low yields.

Next, a one-pot Kostanecki–Robinson cyclization protocol was practiced on the propiophenone 3 in order build the chromone system. The reaction of aromatic ketones with a glycolate ether was considered as an alternative, to access a 2-alkoxymethylene chromone; however, it generally proved to proceed in low yield^11*a*^ and was soon discarded due to inability of the product to withstand the harsh conditions required to break the ether bond and the lack of selectivity of the reaction. In addition, model experiments with the scarcely precedented use of glyoxal^11*b*^ as a two-carbon synthon, which would have delivered a 2-hydroxymethyl chromone directly, met with failure; no reaction was observed, despite different basic conditions (KOH, K^*t*^BuO and NH_4_OH in EtOH) were explored.

Therefore, the ketone 3 was exposed to ethyl chlorooxoacetate,^12^ which can be regarded as an oxidized form of glyoxal or glycolate ethers. Luckily, the reaction took place in CH_2_Cl_2_, in the presence of triethylamine at 100 °C under microwaves irradiation (MW), giving chromone 8 in a reproducible but rather moderate (42%) yield.

Finally, the required adjustment of the oxidation state of the C-2 substituent was carried out by means of a selective reduction of 8; however, since the use of NaBH_4_ as reducing agent^13^ did not perform as expected, this challenging transformation was carried out with Ca(BH_4_)_2_.^14^ The reagent was prepared *in situ* by adding a stoichiometric amount of CaCl_2_ to NaBH_4_ in EtOH.^14*c*^ Under these conditions, chromanone A (1, compound A of Fig. 1) was obtained in 41% yield. Its spectroscopic data in CDCl_3_ confirmed its structure, whereas the NMR spectra taken in MeOH-*d*_4_ were in full agreement with those reported for the natural product.^3^ Signal enhancement (NOE) of the C*H*_2_OH moiety (0.8%) was observed upon irradiation of the hydrogen atoms of the 3-Me group, as well as between the hydrogen atoms of the 8-OMe group and H-7 (1.7%). Notably, the spectra taken in both solvents were alike, and differences exceeding 10 ppm were found between them, particularly for the carbon atoms of the isocyclic ring attached to oxygen.

Despite this first approach to 1 afforded the natural product in just four steps and 13.5% overall yield, the lack of robustness of the selective oxidation of 7, coupled to the moderate yields of the cyclization toward 8 and its selective ester moiety reduction stages, suggested the need to devise an improved alternative.

Therefore, we resorted to the second synthetic plan toward 1, commencing with the selective Friedel–Crafts *ortho*-acylation of pyrocatechol (6) under BF_3_·OEt_2_ promotion.^15^ However, since in our hands the reported procedure proved hard to be reproduced, a series of optimization experiments were run in order to find the proper reaction conditions.

In the process, it was soon found that ZnCl_2_ and AlCl_3_ are not suitable promoters, and that the use of 1,2-dichlorobenzene as solvent negatively affects the reaction performance. It was also discovered that under conventional thermal conditions (Table 1), the transformation hardly proceeded after 3 days at 110 °C (entry 1), whereas only small amounts of product were recovered when the reaction was performed at 180 °C for 5 h (entry 2).

**Table 1:** Optimization of the synthesis of propiophenone 4

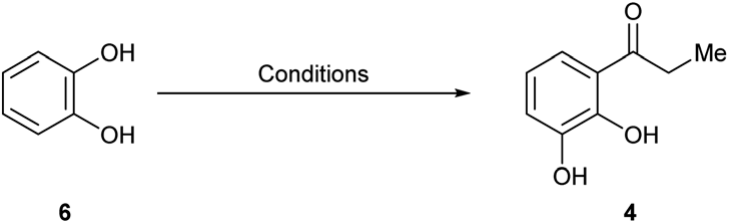						
Entry no.	EtCO_2_H (equiv.)	BF_3_·Et_2_O (equiv.)	Temp. (°C)	Power MW (W)	Time (min)	Yield of 4 (%)
1	9	1	110	—	3 days	7
2	13	1	180	—	300	23
3	9.5	1	180	200	10	0
4	10	1.5	170	100	5	33
5	12	1	170	100	5	61
6	20	1	170	100	5	27
7	12	1	160	100	10	50
8	6.4^a^	1	170	100	5	29

a Propionic anhydride was employed, in the presence of BHT (0.05 equiv.).

On the other hand, employing microwaves radiation, no product was isolated after 10 min at 180 °C (entry 3), whereas slightly milder conditions (170 °C, 5 min) resulted in 33% yield of product (entry 4), when 10 equiv. EtCO_2_H were used. Increasing the amount of acid caused a further increase in the yield to 61% (entry 5), while unexpectedly, additional amounts of acid produced a drastic yield reduction (entry 6). In addition, performing the reaction at 160 °C for 10 min (entry 7) or in the presence of propionic anhydride (entry 8) did not improve the results.

Therefore, the reaction was best performed as in entry 5, in a solventless condition and under microwaves irradiation, affording consistently over 60% yield of the expected product 4.^16^ The use of 6 instead of guaiacol for this approach was based on literature precedents, which suggested that the latter should not be a suitable starting material because it would afford the unwanted propiophenone isomer.^17*a*,*b*^

**Scheme 3:**
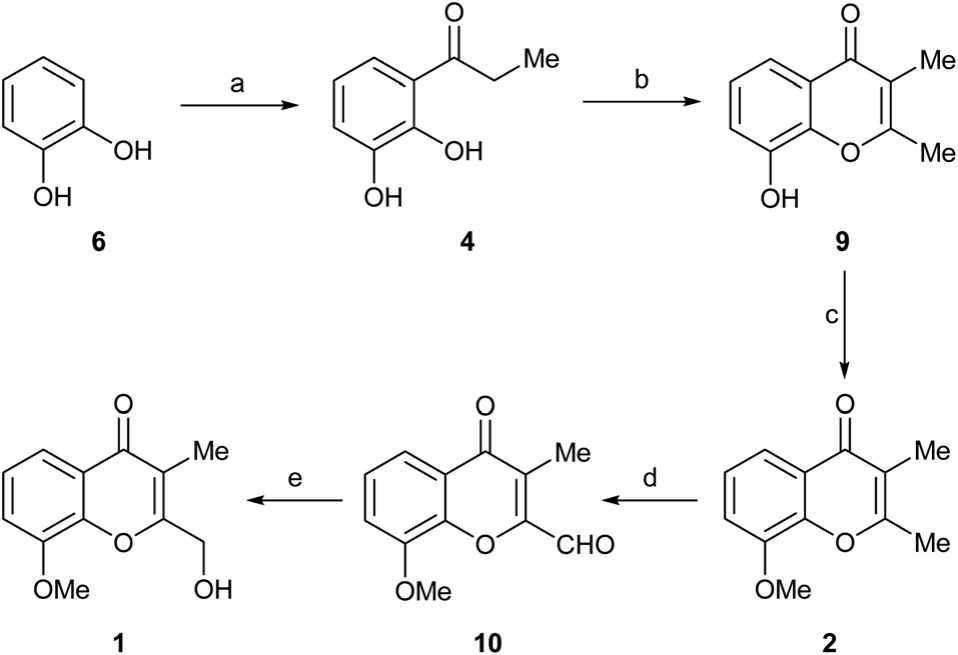
Reagents and conditions: (a) MeCH_2_CO_2_H, BF_3_·OEt_2_, MW (160 °C), 5 min (61%); (b) 1. Ac_2_O, NaOAc, reflux, 3 h; 2. Et_3_N, 115 °C, 14 h; 3. 1 M HCl, 40 °C, 3 h (76% overall); (c) K_2_CO_3_, MeI, Me_2_CO, reflux, 24 h (94%); (d) I_2_, TsOH, DMSO, O_2_, 130 °C, 2 h (83%); (e) NaBH_4_, EtOH, 0 °C → RT, 1 h (70%).

Next, 4 was cyclocondensed and further cyclized with Ac_2_O under Kostanecki-Robinson conditions, uneventfully affording the expected 2,3-dimethylchromone 9 (ref. 7*c*) in 76% yield (Scheme 3). A subsequent Williamson *O*-methylation of 9 with MeI/K_2_CO_3_ in refluxing acetone gave 94% yield of 2.^17*c*^ This was followed by a selective oxidation with the versatile I_2_/DMSO reagent system, which conveniently furnished aldehyde 10 in 83% yield. The transformation, which was carried out aerobically, required the addition of TsOH.^18*a*^

Finally, the reduction of the formyl moiety with NaBH_4_ in EtOH uneventfully provided the expected product 1 in 70% yield. Interestingly, unlike the reduction of compound 8, in this case the use of Ca(BH_4_)_2_ proved to be unnecessary, since the formyl group attached to C-2 is much more reactive than the C-4 ketone moiety.

This optimized approach gave 1 in 25.3% overall yield, after five synthetic steps. The NMR spectroscopic data of the synthetic compound (in MeOH-*d*_4_) were in excellent agreement with those of the literature,^3^ and the heterocycle obtained through Route a, confirming the structure of the natural product.

**Scheme 4:**
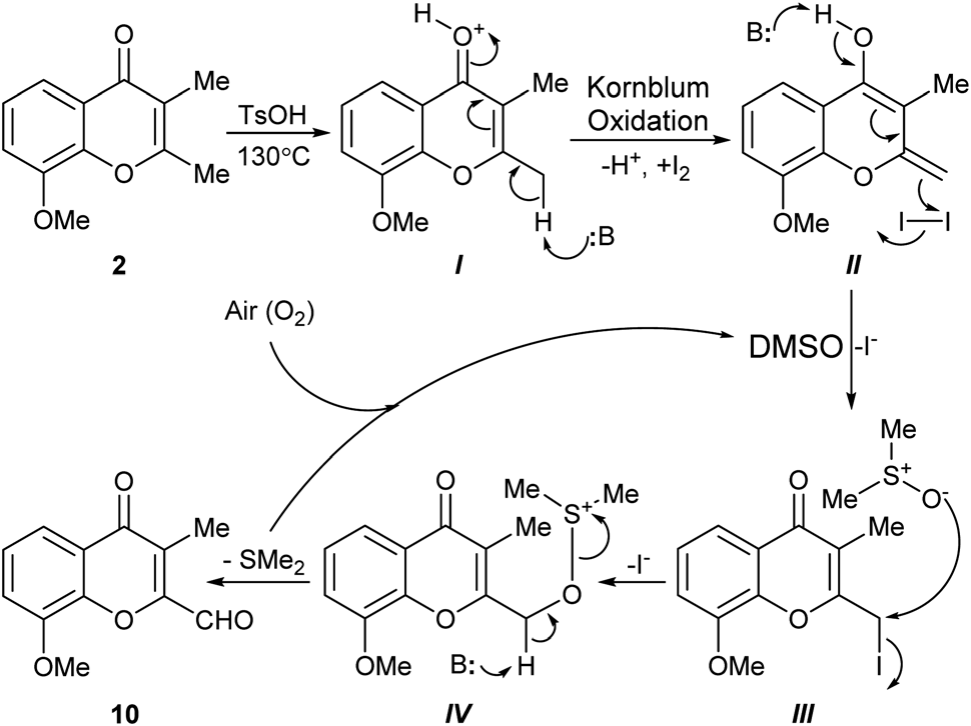
The Kornblum oxidation-based mechanistic proposal for the conversion of 2,3-dimethylchromone 2 into 10 with the I_2_/DMSO reagent system.

The intimate details of the mechanism of the key I_2_/DMSO-mediated oxidation toward 10 remain unclear; however, based on some previous literature precedents, a polar rather than a free-radical reaction mechanism can be proposed. Further, considering that the 2-methylchromone moiety may be regarded as a vinylogous α-methylketone,^18^ it can be conjectured that a Kornblum-like oxidation is at the heart of the mechanistic sequence (Scheme 4). In this scenario, at first the carbonyl group of the substrate (2) would be activated by the added TsOH, giving rise to intermediate I. In turn, this intermediate would be subjected to deprotonation to provide the dienol II, being followed by an iodination with I_2_, to generate the reactive iodide III.

Next, in the presence of DMSO which plays the dual role of solvent and oxidant, the iodide III would undergo a SN_2_-type reaction with the nucleophilic oxygen atom of the reagent, losing iodide and forming the alkoxysulfonium salt intermediate IV. The latter would undergo a proton abstraction, resulting in the aldehyde product 10. Interestingly, it has been reported that reaction of a primary iodide like III with KO_2_ in DMSO afforded a hydroxymethyl chromone, albeit in rather low yield (27%).^19^

During the reaction two molecules of HI and one of SMe_2_ are produced. Although DMSO can oxidize iodide ions to iodine, generating SMe_2_ (DMSO + 2I^−^ → SMe_2_ + I_2_ + H_2_O), it is assumed that the presence of oxygen (air) under the strenuous reaction conditions (130 °C) would serve to reoxidize all the SMe_2_ formed in the transformation and/or to regenerate the iodine in the presence of DMSO.

The general guidelines provided by the retrosynthetic analysis of Scheme 1 were used to synthesize compound 16 (Fig. 1, compound B) and to access additional heterocyclic intermediates for bioactivity testing.

**Scheme 5:**
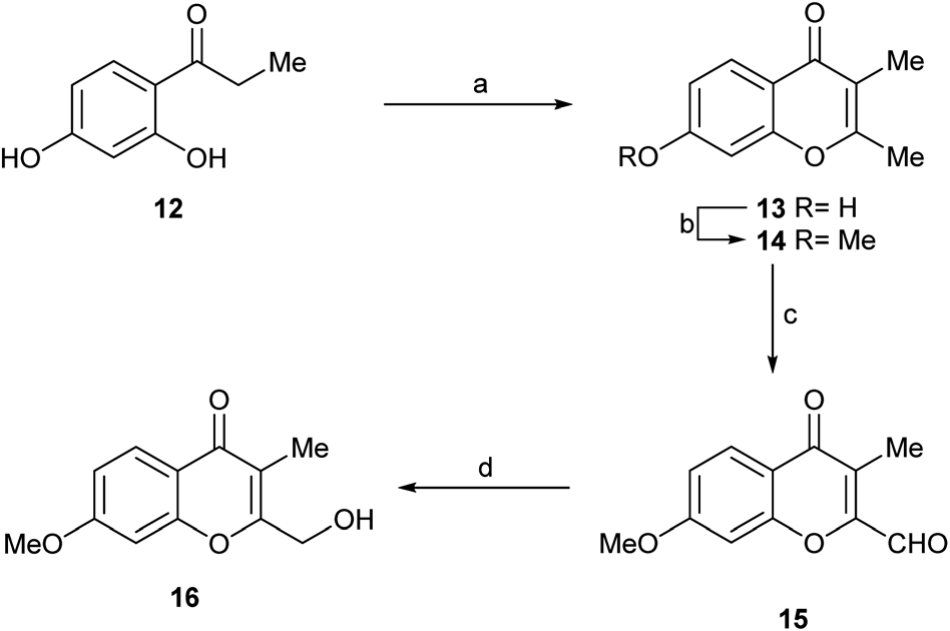
Reagents and conditions: (a) 1. Ac_2_O, NaOAc, reflux, 3 h; 2. Et_3_N, 115 °C, 14 h; 3. 1 M HCl, 40 °C, 6 h (77% overall); (b) MeI, K_2_CO_3_, Me_2_CO, reflux, 24 h (93%); (c) I_2_, TsOH, DMSO, O_2_, 130 °C, 2 h (78%); (d) NaBH_4_, EtOH, 0 °C → RT, 1 h (71%).

The synthetic sequence commenced with the commercially available propiophenone 12 (Scheme 5) which was sequentially exposed to Ac_2_O/NaOAc,^20^ Et_3_N and HCl. Under these conditions, it experienced an exhaustive acetylation, followed by a Baker–Venkataraman rearrangement and cyclization, and a final dehydration and deacetylation to afford 13 in 77% overall yield.

The phenol was methylated under conventional conditions, with MeI in refluxing acetone, using K_2_CO_3_ as base to afford 14 (93%). This is a natural product, which has been recently isolated from *Rhinocladiella* sp.^4*b*^ and from the co-culture of a marine-derived Actinomycete (*Streptomyces rochei* MB037) and the fungus *Rhinocladiella similis* 35. Compound 14 proved to display weak antibacterial activity against *Staphylococcus aureus* and *Pseudomonas aeruginosa*.^21^

Next, 14 was selectively oxidized with the DMSO–I_2_ reagent system, providing aldehyde 15 in 78% yield.^22^ Finally, the aldehyde was selectively reduced with the aid of the NaBH_4_, affording the alcohol 16 (Fig. 1, compound B) in 71% yield. This synthetic route provided the natural product 16 with an overall yield of 39.7% in just four steps.

### 2.2 Evaluation of bioactivity

The methods employed are fully described in the ESI.† Initially, antifungal susceptibility tests were carried out, with the determination of the minimum inhibitory concentration (MIC) and the minimum fungicidal concentration (MFC). The antifungal activity of the compounds was assessed in the concentration range 0.49–250 μg mL^−1^ with the standardized CLSI microbroth dilution method for yeasts^23^ against *C. albicans* ATCC 10231. Initially, the aldehyde 10 and the natural product (1) were screened; however, in order to obtain a better insight into the structural factors implied in the bioactivity, their precursors 2 and 9 as well as the related 7-substituted 2,3-dimethylchromones 13 and 14 were also tested, along with the aldehyde 15 and the natural product 16.

**Fig. 2:**
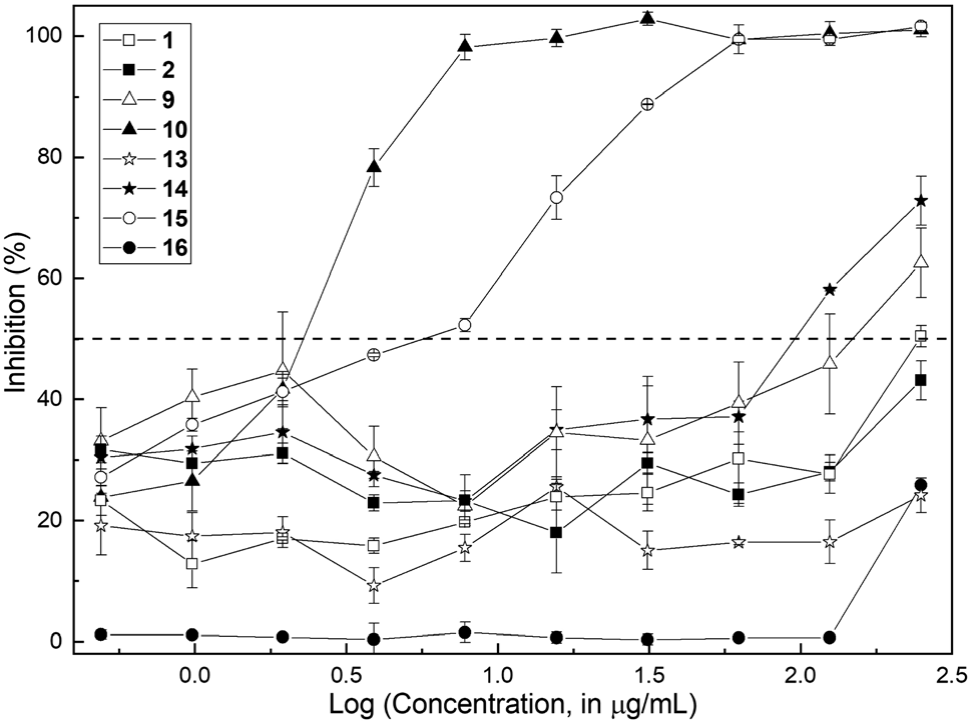
Semi-logarithmic plot of the growth inhibition of *Candida albicans* ATCC 10231 in the presence of different concentrations of the chromone test compounds. The dashed line indicates the 50% inhibition level.

The results are collected in Fig. 2, which depicts the percentage of growth inhibition of a standardized inoculum of *C. albicans* (1 × 10^3^ CFU per mL) plotted against the logarithm of the corresponding concentration of each compound.

The assay revealed that the natural product (1) is a very weak inhibitor, being considered inactive. It barely caused 50% inhibition at a concentration of 250 μg mL^−1^ (MIC > 250 μg mL^−1^); quite a similar profile was exhibited by its phenolic precursor 9 (63% inhibition at 250 μg mL^−1^). On the contrary, the aldehyde 10 proved to be a very good inhibitor with MIC = 7.8 μg mL^−1^.

Regarding the 7-substituted chromones, compounds 13, 14 and 16 demonstrated to be inactive. Compound 14 exhibited 72.8% inhibition at 250 μg mL^−1^ (MIC > 250 μg mL^−1^), whereas aldehyde 15 was moderately active, with a MIC value of 62.5 μg mL^−1^. The MFC values for the isomeric active aldehydes 10 and 15 were also determined according to the established protocol,^23^ being 125 μg mL^−1^ in both cases.

The results suggested that the presence of a formyl moiety is relevant for the observed activity, and that the latter can be modulated by the position of the oxygenated substituent in the homocyclic ring.

Next, the effect of the heterocycles on the virulence factors of *C. albicans* were examined. The tests included inhibition of the adherence to buccal epithelial cells, inhibition of the formation of the germ-tube, morphogenesis of *C. albicans* on solid media and the inhibition of lytic enzymes.

The first step by which a microorganism can initiate an infection is through adherence to an epithelial surface; this ability enables it to exist in biofilms and is in clear association with its virulence.^24^ In order to evaluate whether the active compounds are able to affect this process, at sub-lethal concentrations, compounds 10 and 15 were submitted to the assay of inhibition of the adherence to buccal epithelial cells (BEC).

**Fig. 3:**
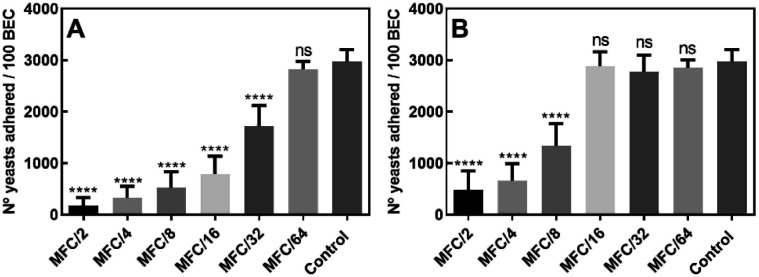
Adherence of *Candida albicans* ATCC 10231 to human buccal epithelial cells (BEC) after incubation in media containing sub-lethal concentrations of 10 (A) and 15 (B). The adherence to BEC is expressed as the number of adhered yeasts/100 BEC. Wilcoxon test = ****(*p* < 0.0001), ns: not significant.

The results (Fig. 3A) showed that the number of yeasts adhered to 100 BEC decreased from 2972 ± 233 in the untreated control cells to 177 ± 60, in the presence of compound 10 at MFC/2; interestingly, the compound was still active at MFC/32 (number of yeasts adhered to 100 BEC = 1724 ± 399). Further, as seen in Fig. 3B, treatment with sub-lethal concentrations of compound 15 also caused a remarkable decrease in yeast adherence to BEC at levels ranging from MFC/2 (482 ± 187) to MFC/8 (1334 ± 233).

These results clearly indicated that the amount of adhered fungal cells to BEC was significantly lower (Wilcoxon test, *p* < 0.0001) in yeasts treated with both aldehydes than in the untreated cells, suggesting that the presence of these heterocycles causes some degree of resistance to the colonization of BEC by *C. albicans*.

In the germ-tube inhibition assay, the effect of different geometrically distributed sub-lethal concentrations (MFC/64–MFC/2) of compounds 10 and 15 on the formation of germ tubes (GT) in *C. albicans* was assessed.

**Fig. 4:**
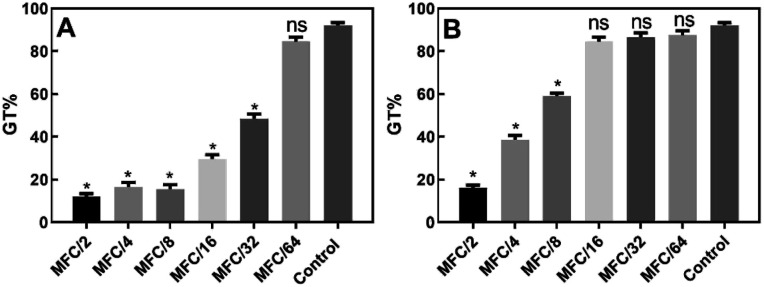
*Candida albicans* ATCC 10231 germ tube formation (% cells) either without (control) or with exposure to sub-lethal concentrations of compounds 10 (A) and 15 (B). Holm–Sidak test = *(*p* < 0.05), ns: not significant.

The results (Fig. 4A) showed that the presence of compound 10 at concentrations between MFC/2 and MFC/32, inhibited the formation of hyphae (GT% = 48.5 ± 2.1% at MFC/32) in a dose-dependent way with respect to the control (GT% = 92 ± 1.4%). The maximum degree of inhibition was recorded at MFC/2 (GT% = 12.0 ± 1.4%).

Compound 15 proved to be a less powerful inhibitor (Fig. 4B), which inhibited hyphae formation up to a concentration of MFC/8 (GT% = 59 ± 1.4%). At MFC/2, the observed GT% was 16.0 ± 1.4%. The results were all statistically significant according to the Holm–Sidak test (*p* < 0.05).

The morphogenesis of *C. albicans* on solid media was studied by examining the effects of compounds 10 and 15 on the formation of pseudomycelium in *C. albicans* in the nutrient-poor Spider medium, that induces pseudohyphal morphogenesis. It was observed that the aldehyde 10 effectively reduced formation of the hyphal pseudomycelium up to a concentration of MFC/4. This effect was apparent by the smoother aspect of the colonies and the notable reduction of hyphae at the edges. On the other hand, in the presence of compound 15, the *C. albicans* colonies showed their typical filamentation at the edges, suggesting that this heterocycle is not an effective inhibitor of pseudomycelium formation.

Finally, in the study of lytic enzyme inhibition, it was detected a statistically significant inhibition of phospholipases secretion in the presence of compound 10 at MFC/2 (Pz = 0.92 ± 0.01) and MFC/4 (Pz = 0.82 ± 0.02) with respect to the control (Pz = 0.74 ± 0.02). Contrarily, however, no significant changes in the Pz index were observed between the untreated (control) cells and those exposed to compound 15. This signaled that the secretion of phospholipases was not inhibited.

On the other hand, it was also observed that at sub-lethal concentrations, compounds 10 and 15 did not inhibit secretion of *C. albicans* esterases, since their values of the Pz index (Pz = 0.82 ± 0.02) were the same as the control.

In view of these promising observations, the interaction of the chromone derivatives on formation of the *C. albicans* biofilm and on preformed biofilms was examined. In the first case, an inoculum prepared according to Pierce *et al.* was used, employing yeast extract–peptone–dextrose (YPD) medium.^25^

**Fig. 5:**
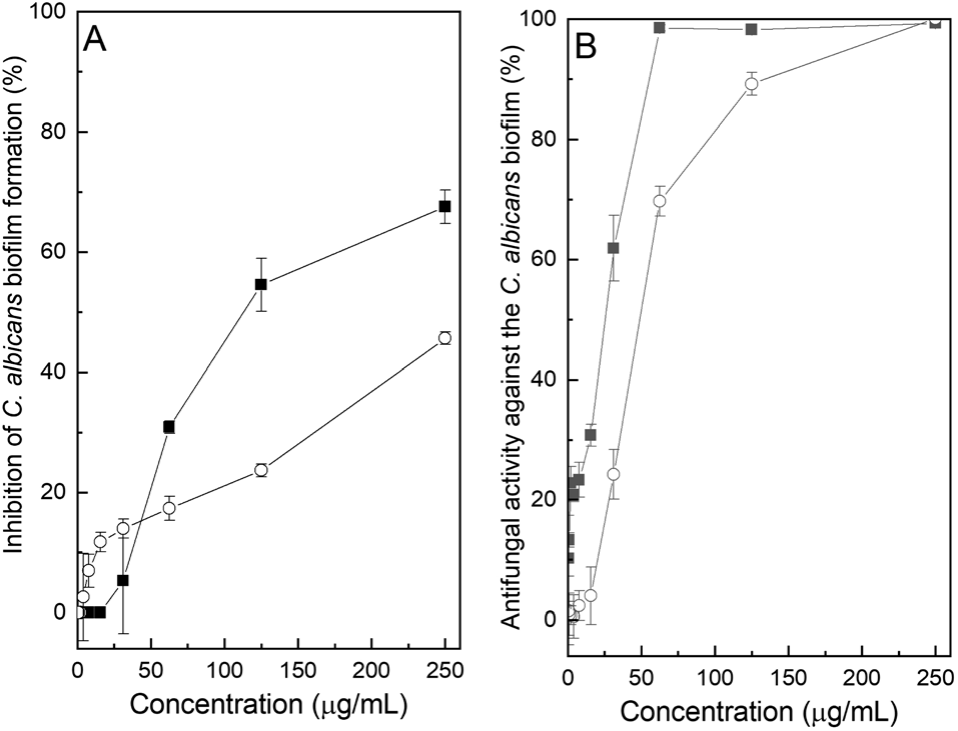
Activity of compounds 10 (■) and 15 (○) against the *C. albicans* ATCC 10231 biofilm. (A) Inhibition of the biofilm formation. (B) Antifungal activity against the pre-formed biofilm.

Fig. 5A displays the data related to the inhibition of the formation of the *C. albicans* biofilm in the presence of different concentrations of compounds 10 and 15. A compound was considered active if it significantly inhibited biofilm formation at concentrations below the MIC (sub-inhibitory). However, it was detected that none of the heterocycles managed to completely avoid formation of the biofilm.

In the presence of 10 at a level of 250 μg mL^−1^, the inhibition was 67.6% (54.5% at 125 μg mL^−1^), while 15 exhibited meager values of 45.6% and 23.7% inhibition at the same concentrations, respectively. Not unexpectedly, at the corresponding MIC values, their degree of inhibition lacked statistical significance; therefore, based on these results, none of the heterocycles inhibited biofilm formation.

On the other hand, when the antifungal activity against preformed *C. albicans* biofilms was evaluated, it was observed that compounds 10 and 15 displayed almost complete inhibition at concentrations of 62.5 and 250 μg mL^−1^, respectively. Further, compound 10 caused over 60% inhibition when the concentration was halved. The effect was concentration dependent, as reflected in the progressive increase in cell viability with reducing concentrations of both compounds.

The results indicate that, while the MIC of compound 10 against planktonic cells of *C. albicans* is 7.8 μg mL^−1^, the aldehyde can completely inhibit the growth of the biofilm yeast colonies at a concentration of 62.5 μg mL^−1^, suggesting that it may be a useful and promising advantage in the development of more effective anti-biofilm agents.

## 3. Conclusions

Two facile and expedient approaches to the first total synthesis of chromanone A, a unique chromone isolated from an algicolous fungus *Penicillium* sp., endophyte of the Egyptian green alga *Ulva* sp., were developed. In the most efficient sequence, the heterocycle was accessed in only five steps and 25.3% overall yield from pyrocatechol, a commercial and easily available starting material.

The key stages of the synthesis included a selective Friedel–Crafts acylation of the starting catechol under BF_3_·Et_2_O promotion and the aerobic oxidation of the C-2 methyl group of a chromone intermediate by the I_2_/DMSO reagent system. To the best of our knowledge, this is the first report of the use of this approach and this reagent system for the synthesis of 2-formyl- and 2-hydroxymethyl chromones. A 7-methoxy isomer of the natural product, which is also a fungal natural product, was prepared following the same strategy. Neither of the synthetic sequences required the use of protecting groups, favouring the efficiency of the syntheses.

The tests of antifungal activity against *C. albicans* revealed that 8-methoxy-3-methyl-4-oxo-4*H*-chromene-2-carbaldehyde, the 2-formyl analogue of chromanone A, exhibited a MIC = 7.8 μg mL^−1^ (MFC = 125 μg mL^−1^). In addition, at sub-lethal concentration levels, this compound significantly inhibited various *C. albicans* virulence factors, including the secretion of phospholipases and the yeast adherence to buccal epithelial cells, as well as the formation of the hyphal pseudomycelium and germ tubes. On the other hand, the formyl derivative completely inhibited the growth of *C. albicans* in preformed biofilms at 62.5 μg mL^−1^.

Taken together, the results indicate that the 2-formyl analogue of chromanone A not only kills *C. albicans*, but also inhibits its virulence factors, suggesting that it is able to target both, the cell growth and the pathogenic process. These features turn the aldehyde into a potentially useful lead to develop more effective agents to fight this yeast in biofilm scenarios. These findings also support the hypothesis that marine fungi may be important sources of inspiration for the development of new agents to combat biofilm-forming microorganisms, and perhaps help to overcome difficulties or failure of current therapy for fungal infections.

## 4. Experimental section

### 4.1 General information

The chemical transformations were carried out under dry argon atmospheres, employing freshly distilled anhydrous solvents and oven-dried glassware. Anhydrous CH_2_Cl_2_ was obtained from an MBraun solvent purifier and dispenser system. Absolute EtOH was prepared by refluxing the commercial solvent over clean Mg/I_2_ and distilling from the resulting magnesium alkoxide. Anhydrous Et_3_N was prepared by distillation of the commercial product from CaH_2_. The anhydrous solvents were stored in dry Young ampoules. The rest of the solvents and reagents were used as received.

The reactions were monitored by TLC, employing aluminium supported silica gel 60 GF_254_ plates run in different hexanes:EtOAc mixtures. The chromatographic spots were revealed by exposure to UV light (254 and 365 nm), followed by spraying with the ethanolic *p*-anisaldehyde/sulfuric acid reagent and gentle heating to improve selectivity.

The flash column chromatographies were developed under positive pressure with slurry-packed silica gel 60 H for thin layer chromatography (particle size < 55 μm), employing gradient of solvent polarity techniques with hexanes:EtOAc.

### 4.2 Equipment

The melting points were determined on an Ernst Leitz Wetzlar model 350 hot-stage microscope and are informed uncorrected. The FT-IR spectra were acquired on a Shimadzu Prestige 21 spectrophotometer, as thin films held between two NaCl disks or as solid dispersions in KBr (solid samples). The NMR spectra were recorded in CDCl_3_ with a Bruker Avance 300 NMR spectrometer (300.13 MHz for ^1^H and 75.48 MHz for ^13^C). The chemical shifts are reported in the *δ* scale, in ppm downfield from TMS (*δ* = 0.0 ppm). The residual solvent peaks of CDCl_3_ (*δ*_H_ = 7.26 ppm, *δ*_C_ = 77.16 ppm), were used as internal references. The scalar coupling constant (*J*) and width and half-height (*w*_1/2_) values are given in Hertz. NOE and 2D NMR experiments (HSQC and HMBC) were also acquired in order to ensure unambiguous signal assignment. The HRMS were obtained with a Bruker MicroTOF-Q II instrument from UMyMFOR (Buenos Aires, Argentina). The microwave-assisted reactions were carried out in a CEM Discover microwave reactor. The light microscopy observations and determinations were performed with an Eclipse E100 (Nikon Corp., Tokyo, Japan) instrument. The microplates were read in a VERSA Max microplate reader (Molecular Devices, Sunnyvale, CA, USA).

### 4.3 2-(1-Hydroxypropyl)-6-methoxyphenol (7)

A stirred solution of *ortho*-vanillin (5, 1500 mg, 9.86 mmol) in anhydrous Et_2_O (10 mL) was purged with nitrogen, cooled to 0 °C in an ice bath and treated dropwise with a solution of EtMgI (2.5 M, 24.6 mmol) in Et_2_O, prepared *in situ* from ethyl iodide and magnesium turnings. The mixture was allowed to warm to room temperature and then additionally stirred for 1 hour. At the end of the reaction (assessed by TLC), a saturated solution of NH_4_Cl was added (10 mL) and the organic products were extracted with CH_2_Cl_2_ (3 × 20 mL). The combined organic extracts were washed with brine (10 mL), dried over anhydrous MgSO_4_, and concentrated under reduced pressure. Chromatography of the residue provided 7 (1742 mg, 97%) as a yellowish oil.^8*b*^ IR (film, *ν̃*): 3443, 2965, 1620, 1593, 1487 and 1454 cm^−1^. ^1^H NMR (300 MHz, CDCl_3_): *δ* = 6.85–6.75 (3H, m, H-3, H-4, H-5), 6.31 (1H, s, OH), 4.79 (1H, q, *J* = 6.3, H-1′), 3.90 (3H, s, OMe), 2.55 (1H, d, *J* = 5.6, OH) 1.97–1.78 (2H, m, H-2′) and 0.96 (3H, t, *J* = 7.4, Me). ^13^C NMR (75 MHz, CDCl_3_): *δ* = 146.8 (C-6), 143.1 (C-1), 129.5 (C-2), 119.6 (C-3)*, 119.3 (C-4)*, 109.8 (C-5), 73.4 (C-1′), 56.1 (OMe), 30.1 (C-2′) and 10.3 (C-Me).

### 4.4 1-(2-Hydroxy-3-methoxyphenyl)propan-1-one (3)

A stirred solution of 7 (34 mg, 0.19 mmol) in EtOAc (1 mL) was treated with 2-iodoxybenzoic acid (78 mg, 0.28 mmol) and the mixture was warmed to 80 °C for 1 h. Once the reaction was completed (after TLC monitoring), the solvent was removed under reduced pressure and the resulting crude was chromatographed, affording 3 (27.4 mg, 81%) as a yellowish solid,^26^ mp: 69–71 °C (Lit.: 72–73 °C).^10^ IR (KBr, *ν̃*): 3420, 2918, 1636, 1458, 1435, 1368, 1317, 1273, 1252, 1227, 1088, 1022, 986, 822, 806, 770, 739, 719 and 625 cm^−1^. ^1^H NMR (300 MHz, CDCl_3_): *δ* = 7.37 (1H, dd, *J* = 1.5, 8.0, H-6′), 7.05 (1H, d, *J* = 8.0, H-4′), 6.84 (1H, t, *J* = 8.0, H-5′), 3.91 (3H, s, OMe), 3.05 (2H, q, *J* = 7.4, H-2) and 1.24 (3H, t, *J* = 7.4, Me). ^13^C NMR (75 MHz, CDCl_3_): *δ* = 207.5 (C-1), 152.8 (C-2′), 149.0 (C-3′), 121.0 (6′), 119.3 (C-4′), 118.2 (C-5′), 116.7 (C-1′), 56.2 (OMe), 31.9 (C-2) and 8.2 (Me). HRMS (ESI^+^) calcd for C_10_H_13_O_3_ [M^+^]: 181.0859, found 181.0859.

### 4.5 Ethyl 8-methoxy-3-methyl-4-oxo-4*H*-chromene-2-carboxylate (8)

Under an argon atmosphere, anhydrous triethylamine (198 mg, 1.95 mmol) and ethyl chlorooxoacetate (67 mg, 0.49 mmol) were successively added to a stirred solution of 3 (44 mg, 0.24 mmol) in anhydrous CH_2_Cl_2_ (1 mL), placed in a microwave vial. The mixture was heated at 100 °C in a microwave oven for 1 h; after its completion was confirmed by TLC, it was cooled to room temperature and diluted with brine (10 mL). The reaction products were extracted with EtOAc (3 × 20 mL). The combined organic extracts were washed with brine (5 mL), dried over MgSO_4_ and concentrated under reduced pressure. The resulting residue was subjected to chromatography, giving 8 (26 mg, 42%) as a yellowish oil. ^1^H NMR (300 MHz, CDCl_3_): *δ* = 7.75 (1H, dd, *J* = 1.5, 8.0, H-5), 7.32 (1H, t, *J* = 8.0, H-6), 7.18 (1H, dd, *J* = 1.5, 8.0, H-7), 4.49 (2H, q, *J* = 7.2, CH_2_Me), 4.01 (3H, s, OMe), 2.37 (3H, s, Me) and 1.46 (3H, t, *J* = 7.2, CH_2_*Me*). ^13^C NMR (75 MHz, CDCl_3_): *δ* = 178.8 (C-4), 161.8 (*C*O_2_Et), 148.9 (C-2), 148.7 (C-8), 146.0 (C-8a), 125.1 (C-6), 123.9(C-3), 123.5 (C-4a), 116.7 (C-5), 114.7 (C-7), 62.6 (*C*H_2_Me), 56.5 (OMe), 14.1 (CH_2_*Me*) and 10.3 (Me-3). HRMS (ESI^+^) calcd for C_14_H_15_O_5_ [M^+^]: 263.0914, found 263.0911.

### 4.6 2-(Hydroxymethyl)-8-methoxy-3-methyl-4*H*-chromen-4-one (1)

A cold (5 °C) mixture of CaCl_2_ (13.0 mg, 0.11 mmol) and NaBH_4_ (3.5 mg, 0.09 mmol) in an EtOH : THF (1 : 1 v/v, 500 μL) solvent mixture was added to a stirred solution of 8 (20 mg, 0.08 mmol) in EtOH : THF (1 : 1 v/v, 500 μL), placed in an ice-water bath. After 24 h, no starting material was observed by TLC and the reaction was quenched with acetone (1 mL), the solvents were removed under reduced pressure and the crude product was chromatographed, yielding 1 (6.8 mg, 41%) as a yellow solid, mp: 152–154 °C. IR (KBr, *ν̃*): 3397, 2955, 2916, 2849, 1734, 1717, 1684, 1636, 1609, 1570, 1497, 1458, 1398, 1375, 1362, 1285, 1265, 1225, 1179, 1163, 1115, 1072 and 1020 cm^−1^. ^1^H NMR (300 MHz, CDCl_3_): *δ* = 7.76 (1H, dd, *J* = 1.4, 8.0, H-5), 7.29 (1H, t, *J* = 8.0, H-6), 7.12 (1H, dd, *J* = 1.4, 8.0, H-7), 4.75 (2H, s, H-1′), 3.98 (3H, s, OMe), 2.65 (1H, bs, *w*_1/2_ = 13, OH), and 2.12 (3H, s, Me-3). ^13^C NMR (75 MHz, CDCl_3_): *δ* = 178.3 (C-4), 160.5 (C-2), 148.5 (C-8), 146.2 (C-8a), 124.5 (C-6), 123.6 (C-4a), 117.4 (C-3), 116.8 (C-5), 113.8 (C-7), 60.4 (CH_2_), 56.2 (OMe) and 9.2 (Me-3).

^1^H NMR (300 MHz, MeOH-*d*_4_): *δ* = 8.00 (1H, dd, *J* = 1.5, 7.8, H-5), 7.03 (1H, bs, H-6), 7.02 (1H, dd, *J* = 1.5, 7.8, H-7), 4.62 (2H, s, H-1′), 3.98 (3H, s, OMe) and 2.07 (3H, s, Me). ^13^C NMR (75 MHz, MeOH-*d*_4_): *δ* = 180.1 (C-4), 166.0 (C-8a), 164.2 (C-8), 159.4 (C-2), 127.7 (C-6), 118.1 (C-4), 117.3 (C-4a), 116.0 (C-7), 101.0 (C-3), 60.7 (CH_2_), 56.5 (OMe) and 9.2 (Me-3). The NMR signals of the synthetic compound in MeOH-*d*_4_ were in agreement with those of the natural product (compound A).^3^

### 4.7 1-(2,3-Dihydroxyphenyl)propan-1-one (4)

A mixture of pyrocatechol (6, 100 mg, 0.91 mmol), BF_3_OEt (128.9 mg, 0.91 mmol) and propionic acid (807 mg, 10.9 mmol) reacted together under microwave irradiation at 160 °C, without any solvent for a short time. After cooling to room temperature, the reaction mixture was dissolved in dichloromethane (10 mL) and brine (about 20 mL). After extraction of the organic phase, it was washed with aqueous NaHCO_3_ (20 mL), dried with MgSO_4_, filtered and evaporated to give a crude product. The crude product was chromatographed to afford 11 (92 mg, 61%), as a yellow solid, mp: 54–56 °C (Lit.: 56–57 °C).^16*a*^ IR (KBr, *ν̃*): 3566, 3491, 2978, 2940, 1643, 1636, 1597, 1456, 1452, 1369, 1271, 1109, 1076, 1045, 880, 820 and 731 cm^−1^. ^1^H NMR (300 MHz, CDCl_3_): *δ* = 7.31 (1H, dd, *J* = 1.4, 8.2, H-6′), 7.12 (1H, dd, *J* = 1.4, 7.9, H-4′), 6.81 (1H, t, *J* = 8.0, H-5′), 5.75 (2H, bs, *w*_1/2_ = 76, 2 × OH). 3.04 (2H, q, *J* = 7.3, CH_2_) and 1.24 (3H, t, *J* = 7.3, Me). ^13^C NMR (75 MHz, CDCl_3_): *δ* = 207.7 (C-1), 149.5 (C-2′), 145.5 (C-3′), 120.5 (C-6′), 120.0 (C-4′), 119.1 (C-1′), 118.9 (C-5′), 31.7 (CH_2_) and 8.2 (Me).

### 4.8 8-Hydroxy-2,3-dimethyl-4*H*-chromen-4-one (9)

A solution of 4 (110 mg, 0.66 mmol) in Ac_2_O (2973 mg, 29.13 mmol) was treated with anhydrous NaOAc (271 mg, 3.31 mmol) and the mixture was heated under reflux for 3 h. The excess Ac_2_O was distilled under reduced pressure (6 mm Hg), the residue was suspended with AcOEt and the remainder of NaOAc was filtered off under reduced pressure, supplying a mixture of acetates. The acetates were treated with anhydrous Et_3_N (0.5 mL) and the mixture was heated for 14 h at 115 °C. After that period, the reaction was cooled to room temperature, the solvent was removed under reduced pressure and the residue was treated with 1 M HCl solution at 5 °C (5 mL). The resulting suspension was stirred for 6 h at 40 °C and the white precipitate formed was filtered under reduced pressure and washed with water at 5 °C. Chromatography of the product provided 9 (96.1 mg, 76% overall), as a yellowish solid, mp: 195–197 °C (Lit.: 203–204 °C).^27^ IR (KBr, *ν̃*): 3462, 3447, 3416, 1636, 1616, 1578, 1558, 1362, 1269, 1159, 1134, 762 and 619 cm^−1^. ^1^H NMR (300 MHz, (CD_3_)_2_CO): *δ* = 9.05 (1H, s, OH), 7.59–7.51 (1H, m H-5), 7.26–7.19 (2H, m, H-6, H-7), 2.45 (3H, s, Me-3) and 2.00 (3H, s, Me-2). ^13^C NMR (75 MHz, (CD_3_)_2_CO_3_): *δ* = 177.5 (C-4), 162.3 (C-2), 147.0 (C-8), 146.2 (C-8a), 125.2 (C-6), 124.5 (C-4a), 118.9 (C-7), 117.1 (C-3), 116.1 (C-5), 18.3 (Me-2) and 10.0 (Me-3). HRMS (ESI^+^) calcd for C_11_H_11_O_3_ [M^+^]: 191.0703, found 191.0705.

### 4.9 8-Methoxy-2,3-dimethyl-4*H*-chromen-4-one (2)

A solution of 9 (69 mg, 0.36 mmol) and K_2_CO_3_ (70,2 mg, 0.51 mmol) in anhydrous acetone (1 mL) was heated under reflux and treated with MeI (66.9 mg, 0.47 mmol) for 24 h. Then the solvent was evaporated, brine (10 mL) added, and the product was extracted with EtOAc (3 × 20 mL). The combined extracts were concentrated under reduced pressure and the residue was chromatographed to afford 2 (70 mg, 94%), as a white solid, mp: 149–151 °C (Lit.: 154 °C).^17*c*^^1^H NMR (300 MHz, CDCl_3_): *δ* = 7.72 (1H, dd, *J* = 1.4, 8.1, H-5), 7.22 (1H, t, *J* = 8.0, H-6), 7.07 (1H, dd, *J* = 1.4, 7.9, H-7), 3.95 (3H, s, OMe), 2.43 (3H, s, Me-3) and 2.04 (3H, s, Me-2). ^13^C NMR (75 MHz, CDCl_3_): *δ* = 177.7 (C-4), 161.6 (C-2), 148.3 (C-8a), 146.3 (C-8), 124.1 (C-6), 123.6 (C-4a), 117.0 (C-3), 116.8 (C-5), 113.4 (C-7), 56.2 (OMe), 18.6 (Me-2) and 10.1 (Me-3). HRMS (ESI^+^) calcd for C_12_H_13_O_3_ [M^+^]: 205.0859, found 205.0859.

### 4.10 8-Methoxy-3-methyl-4-oxo-4*H*-chromene-2-carbaldehyde (10)

A mixture of 2 (52.9 mg, 0.26 mmol), I_2_ (13.15 mg, 0.10 mmol), TsOH (44.6 mg, 0.26 mmol) in DMSO (1 mL) was heated at 130 °C under oxygen atmosphere for 2 h. After this time, the reaction solution was washed with saturated NH_4_Cl (5 mL) and extracted with CH_2_Cl_2_ (3 × 5 mL), the organic phase was dried with MgSO_4_, filtered and the remaining DMSO was distilled under reduced pressure evaporated to give a crude product. The crude product was chromatographed to afford 10 (47 mg, 83%), as a yellow solid, mp: 150–152 °C. IR (KBr, *ν̃*): 3539, 3420, 3233, 1724, 1636, 1622, 1605, 1582, 1493, 1458, 1441, 1406, 1377, 1356, 1271, 1227, 1194, 1159, 1121, 1076, 1001, 918, 824 and 752 cm^−1^. ^1^H NMR (300 MHz, CDCl_3_): *δ* = 10.20 (1H, s, CHO), 7.73 (1H, dd, *J* = 1.2, 8.4, H-5), 7.33 (1H, t, *J* = 8.1, H-6), 7.20 (1H, dd, *J* = 1.4, 8.0, H-7), 4.01 (3H, s, OMe) and 2.43 (3H, s, Me-2). ^13^C NMR (75 MHz, CDCl_3_): *δ* = 186.5 (C-1′), 179.2 (C-4), 150.5 (C-2), 149.1 (C-8), 145.9 (C-8a), 125.3 (C-6, C-3), 123.6 (C-4a), 116.6 (C-5), 114.9 (C-7), 56.4 (OMe) and 8.2 (Me-2). HRMS (ESI^+^) calcd for C_12_H_11_O_4_ [M^+^]: 219.0652, found 219.0652.

### 4.11 2-(Hydroxymethyl)-8-methoxy-3-methyl-4*H*-chromen-4-one (1)

To a solution of 13 (24 mg, 0.11 mmol) in EtOH (1 mL) was added NaBH_4_ (5.0 mg, 0.13 mmol) under an argon atmosphere at 0 °C and stirred the reaction mixture at RT for 1 h. After completion of the reaction, the reaction mixture was quenched with acetone (1 mL). Then, acetone was distilled under reduced pressure evaporated and the crude product was chromatographed to afford chromanone A (1, 17 mg, 70%), as a yellow solid. The NMR spectroscopic data of this compound exhibited concordance with those recorded for compound A^3^ (Fig. 1) and for 1 when obtained from 5.

### 4.12 7-Hydroxy-2,3-dimethyl-4*H*-chromen-4-one (13)

A solution of 2,4-dihydroxypropiophenone (12, 1000 mg, 6.0 mmol) in Ac_2_O (25 mL, 265 mmol) was treated with anhydrous NaOAc (2468 mg, 30.1 mmol) and the mixture was heated under reflux for 3 h. The excess Ac_2_O was distilled under reduced pressure (6 mm Hg), the residue was suspended with AcOEt and the remainder of NaOAc was filtered off under reduced pressure, supplying a mixture of acetates. The acetates were treated with anhydrous Et_3_N (1 mL) and the mixture was heated for 14 h at 115 °C. After that period, the reaction was cooled to room temperature, the solvent was removed under reduced pressure and the residue was treated with 1 M HCl solution at 5 °C (40 mL). The resulting suspension was stirred for 6 h at 40 °C and the precipitate formed was filtered under reduced pressure and washed with water at 5 °C. Chromatography of the product provided 13 (880 mg, 77% overall) as a yellowish solid, mp: 280–282 °C (Lit.: 281 °C).^28^ IR (KBr, *ν̃*): 3993, 2924, 2853, 1653, 1558, 1541, 1458, 1246, 1101, 860 and 689 cm^−1^. ^1^H NMR (300 MHz, acetone-*d*_6_): *δ* = 9.7 (1H, bs, *w*_1/2_ = 42, OH), 7.94 (1H, d, *J* = 8.7, H-5), 6.81 (1H, dd, *J* = 2.2, 8.7, H-6), 6.71 (1H, d, *J* = 2.2, H-8), 2.31 (3H, d, *J* = 0.6, Me-2) and 1.96 (3H, d, *J* = 0.6, Me-3). ^13^C NMR (75 MHz, acetone-*d*_6_): *δ* = 177.4 (C-4), 163.2 (C-7), 162.3 (C-2), 158.5 (C-8a), 127.9 (C-5), 116.6 (C-3), 116.4 (C-4a), 115.3 (C-6), 102.7 (C-8), 18.4 (Me-2) and 9.9 (Me-3).^7*c*^

### 4.13 7-Methoxy-2,3-dimethyl-4*H*-chromen-4-one (14)

A solution of 13 (800 mg, 4.21 mmol) and K_2_CO_3_ (813.8 mg, 5.89 mmol) in anhydrous acetone (20 mL) was heated under reflux and treated with MeI (776 mg, 5.47 mmol) for 24 h. Then the solvent was evaporated, brine added, and the product was extracted with EtOAc. The combined extracts were concentrated under reduced pressure and the residue was chromatographed to afford 14 (795 mg, 93%) as a as a yellowish solid,^21^ mp: 125–127 (Lit.: 127 °C).^29^ IR (KBr, *ν̃*): 3420, 2924, 1636, 1605, 1443, 1404, 1352, 1244, 1204, 1111, 1030, 856, 824 and 689 cm^−1^. ^1^H NMR (300 MHz, CDCl_3_): *δ* = 8.09 (1H, d, *J* = 8.8, H-5), 6.92 (1H, dd, *J* = 2.5, 8.8, H-6), 6.77 (1H, d, *J* = 2.5, H-8), 3.88 (3H, s, OMe), 2.38 (3H, d, *J* = 0.5, Me-3) and 2.04 (3H, d, *J* = 0.5, Me-2). ^13^C NMR (75 MHz, CDCl_3_): *δ* = 177.4 (C-4), 163.5 (C-7), 161.2 (C-2), 157.6 (C-8a), 127.2 (C-5), 116.6 (C-3), 116.6 (C-4a), 113.9 (C-6), 99.7 (C-8), 55.7 (OMe), 18.4 (Me-2) and 10.0 (Me-3).

### 4.14 7-Methoxy-3-methyl-4-oxo-4*H*-chromene-2-carbaldehyde (15)

A mixture of 14 (20.4 mg, 0.10 mmol), I_2_ (5.07 mg, 0.04 mmol) and TsOH (17.2 mg, 0.10 mmol) was heated at 130 °C under an oxygen atmosphere for 3 h in DMSO (1 mL). After this time, the reaction was diluted with saturated NH_4_Cl solution (10 mL) and the product was extracted with CH_2_Cl_2_ (3 × 10 mL). The combined organic phases were dried with MgSO_4_, and concentrated under reduced pressure. The remaining DMSO was distilled under vacuum and the crude product was chromatographed to afford 15 (17 mg, 78%) as a yellowish solid,^22^ mp: 118–121 °C. IR (KBr, *ν̃*): 2920, 2849, 1699, 1624, 1443, 1281, 1261, 1206, 1159, 1111, 1020, 851, 768 and 623 cm^−1^. ^1^H NMR (300 MHz, CDCl_3_): *δ* = 10.20 (1H, s, CHO), 8.11 (1H, d, *J* = 8.9, H-5), 6.99 (1H, dd, *J* = 2.4, 8.9, H-6), 6.93 (1H, d, *J* = 2.4, H-8), 3.92 (3H, s, OMe) and 2.44 (3H, s, Me-3). ^13^C NMR (75 MHz, CDCl_3_): *δ* = 185.7 (C-1′), 178.3 (C-4), 165.1 (C-7), 157.2 (C-8a), 150.6 (C-2), 127.4 (C-5), 126.6 (C-3), 116.8 (C-4a), 115.7 (C-6), 99.9 (C-8), 55.9 (OMe) and 8.0 (Me-3).

### 4.15 2-(Hydroxymethyl)-7-methoxy-3-methyl-4*H*-chromen-4-one (16)

A stirred solution of 13 (10.5 mg, 0.05 mmol) in EtOH (1 mL) under an argon atmosphere was cooled to 0 °C and treated with NaBH_4_ (2.73 mg, 0.07 mmol). The reaction mixture was further stirred at RT for 1 h, when it was quenched with acetone (1 mL). The volatiles were removed under reduced pressure and the crude product was chromatographed to afford 13 (7.5 mg, 71%), as a white solid, mp: 122–125 °C. IR (KBr, *ν̃*): 3323, 2924, 2853, 1638, 1597, 1449, 1244, 1177, 1042, 1024 and 827 cm^−1^. ^1^H NMR (300 MHz, CDCl_3_): *δ* = 8.06 (1H, d, *J* = 8.9, H-5), 6.92 (1H, dd, *J* = 2.4, 8.9, H-6), 6.74 (1H, d, *J* = 2.4, H-8), 4.67 (2H, s, H-1′), 3.87 (3H, s, OMe), 2.75 (1H, bs, *w*_1/2_ = 16, OH) and 2.07 (3H, s, Me-3). ^13^C NMR (75 MHz, CDCl_3_): *δ* = 178.0 (C-4), 163.9 (C-7), 160.5 (C-2), 157.5 (C-8a), 127.2 (C-5), 117.3 (C-3), 116.4 (C-4a), 114.5 (C-6), 99.6 (C-8), 60.5 (C-1′), 55.7 (OMe) and 9.2 (Me-3). The spectroscopic data of this compound were in full agreement with those recorded for the natural product (compound C).^4*b*^

## Author contributions

All the authors were involved in the manuscript, performing conceptualization (IC, TSK, ABJB, LAS), data curation (IC, MAS, LAS), formal analysis (IC, EC, TSK, ABJB), funding acquisition (TSK, ABJB, MAS, LAS), investigation (IC, EC, TSK, ABJB), methodology (IC, EC, TSK, LAS, MAS, ABJB), project administration (TSK, ABJB, LAS, MAS), resources (TSK, ABJB, LAS, MAS), use of software (IC, TSK, ABJB), supervision (ABJB, TSK, MAS, LAS), results validation (TSK), results visualization (IC, EC, TSK), writing – original draft (IC, EC, TSK, ABJB) and writing – review & editing (IC, EC, TSK, LAS, MAS, ABJB).

## Conflicts of interest

There are no conflicts to declare.

## Supplementary Material

RA-011-D1RA02553H-s001
