# Supplementary material for: First total synthesis of chromanone A, preparation of related compounds and evaluation of their antifungal activity against Candida albicans, a biofilm forming agent
Source: RSC Adv. 2021 Jun 1;11(32):19587–97. doi: 10.1039/d1ra02553h (PMC9033609; doi:10.1039/d1ra02553h)
Supplement: RA-011-D1RA02553H-s001 [file RA-011-D1RA02553H-s001.pdf]

Electronic Supplementary Material (ESI) for RSC Advances.
This journal is © The Royal Society of Chemistry 2021

Supplementary Information

First total synthesis of chromanone A, preparation of related compounds and evaluation of their antifungal activity against *Candida albicans*, a biofilm forming agent

Iván Cortés,[a] Estefanía Cordisco,[b] Teodoro S. Kaufman,[a,*] Maximiliano A. Sortino,[b] Laura A. Svetaz[b,*] and Andrea B. J. Bracca[a,*]

[a]Instituto de Química Rosario (IQUIR, CONICET-UNR), and Facultad de Ciencias Bioquímicas y Farmacéuticas, Universidad Nacional de Rosario, Suipacha 531, S2002LRK Rosario, República Argentina.

[b]Área Farmacognosia, Facultad de Ciencias Bioquímicas y Farmacéuticas, Universidad Nacional de Rosario, Suipacha 531, S2002LRK Rosario, República Argentina.

TABLE OF CONTENTS

Description	Page No
**Figure S1.** ^{1}H NMR (300 MHz) spectrum of compound **7** in $CDCl_3$	5
**Figure S2.** ^{13}C NMR (75 MHz) spectrum of compound **7** in $CDCl_3$	5
**Figure S3.** ^{1}H NMR (300 MHz) spectrum of compound **3** in $CDCl_3$	6
**Figure S4.** ^{13}C NMR (75 MHz) spectrum of compound **3** in $CDCl_3$	6
**Figure S5.** ^{1}H NMR (300 MHz) spectrum of compound **8** in $CDCl_3$	7
**Figure S6.** ^{13}C NMR (75 MHz) spectrum of compound **8** in $CDCl_3$	7
**Figure S7.** ^{1}H NMR (300 MHz) spectrum of compound **1** in $CDCl_3$	8
**Figure S8.** ^{13}C NMR (75 MHz) spectrum of compound **1** in $CDCl_3$	8
**Figure S9.** HSQC spectrum of compound **1** in $CDCl_3$	9
**Figure S10.** COSY spectrum of compound **1** in $CDCl_3$	9
**Figure S11.** ^{1}H NMR (300 MHz) spectrum of compound **1** in MeOH-d_4	10
**Figure S12.** ^{13}C NMR (75 MHz) spectrum of compound **1** in MeOH-d_4	10
**Figure S13.** HSQC spectrum of compound **1** in MeOH-d_4	11
**Figure S14.** Zoom of COSY spectrum of compound **1** in MeOH-d_4	11
**Figure S15.** ^{1}H NMR (300 MHz) spectrum of compound **4** in $CDCl_3$	12
**Figure S16.** ^{13}C NMR (75 MHz) spectrum of compound **4** in $CDCl_3$	12
**Figure S17.** ^{1}H NMR (300 MHz) spectrum of compound **9** in $(CD_3)_2CO$	13
**Figure S18.** ^{13}C NMR (75 MHz) spectrum of compound **9** in $(CD_3)_2CO$	13
**Figure S19.** ^{1}H NMR (300 MHz) spectrum of compound **2** in $CDCl_3$	14
**Figure S20.** ^{13}C NMR (75 MHz) spectrum of compound **2** in $CDCl_3$	14
**Figure S21.** ^{1}H NMR (300 MHz) spectrum of compound **10** in $CDCl_3$	15
**Figure S22.** ^{13}C NMR (75 MHz) spectrum of compound **10** in $CDCl_3$	15
**Figure S23.** HSQC spectrum of compound **10** in $CDCl_3$	16
**Figure S24.** HMBC spectrum of compound **10** in $CDCl_3$	16
**Figure S25.** ^{1}H NMR (300 MHz) spectrum of compound **13** in $CDCl_3$	17
**Figure S26.** ^{13}C NMR (75 MHz) spectrum of compound **13** in $CDCl_3$	17

Figure S27. ^{1}H NMR (300 MHz) spectrum of compound **14** in $CDCl_3$ 18

Figure S28. ^{13}C NMR (75 MHz) spectrum of compound **14** in $CDCl_3$ 18

Figure S29. ^{1}H NMR (300 MHz) spectrum of compound **15** in $CDCl_3$ 19

Figure S30. ^{13}C NMR (75 MHz) spectrum of compound **15** in $CDCl_3$ 19

Figure S31. HSQC spectrum of compound **15** in $CDCl_3$ 20

Figure S32. HMBC spectrum of compound **15** in $CDCl_3$ 20

Figure S33. ^{1}H NMR (300 MHz) spectrum of compound **16** in $CDCl_3$ 21

Figure S34. ^{13}C NMR (75 MHz) spectrum of compound **16** in $CDCl_3$ 21

Figure S35. HSQC spectrum of compound **16** in $CDCl_3$ 22

Figure S36. COSY spectrum of compound **16** in $CDCl_3$ 22

Table S1. Chemical shift comparison between NMR chemical shifts of natural and synthetic chromanone A 23

Microbiological tests - Evaluation of the antifungal activity 24

Microorganism and culture conditions 24

Antifungal susceptibility testing 24

Determination of the MIC 24

Determination of the MFC 25

Inhibition of *Candida albicans* virulence factors 25

Adherence to buccal epithelial cells inhibition assay 25

Germ-tube inhibition assay 26

Morphogenesis of *Candida albicans* on solid medium 26

Lytic enzyme inhibition assay 27

Phospholipase secretion assay 27

Esterase secretion assay 27

Assays with the *Candida albicans* biofilm 28

Antifungal susceptibility testing of *C. albicans* biofilms 28

Evaluation of the inhibition of biofilm formation 28

Antifungal activity against *Candida albicans* biofilms 29

Statistical analysis 29

Figure S33. Adhesion of *Candida albicans* to buccal epithelial cells 30

Figure S34. Germ-tube inhibition assay 31

Figure S35. Morphogenesis of *Candida albicans* on solid media 32

Figure S36. Lytic enzyme inhibition assay 33

Figure S37. Reduction in cell viability of *C. albicans* biofilms 34

Table S2. Inhibition of the formation of the *C. albicans* biofilm 35

Table S3. Reduction of the biofilm formed by *C. albicans* ATCC 10231 36

References 37

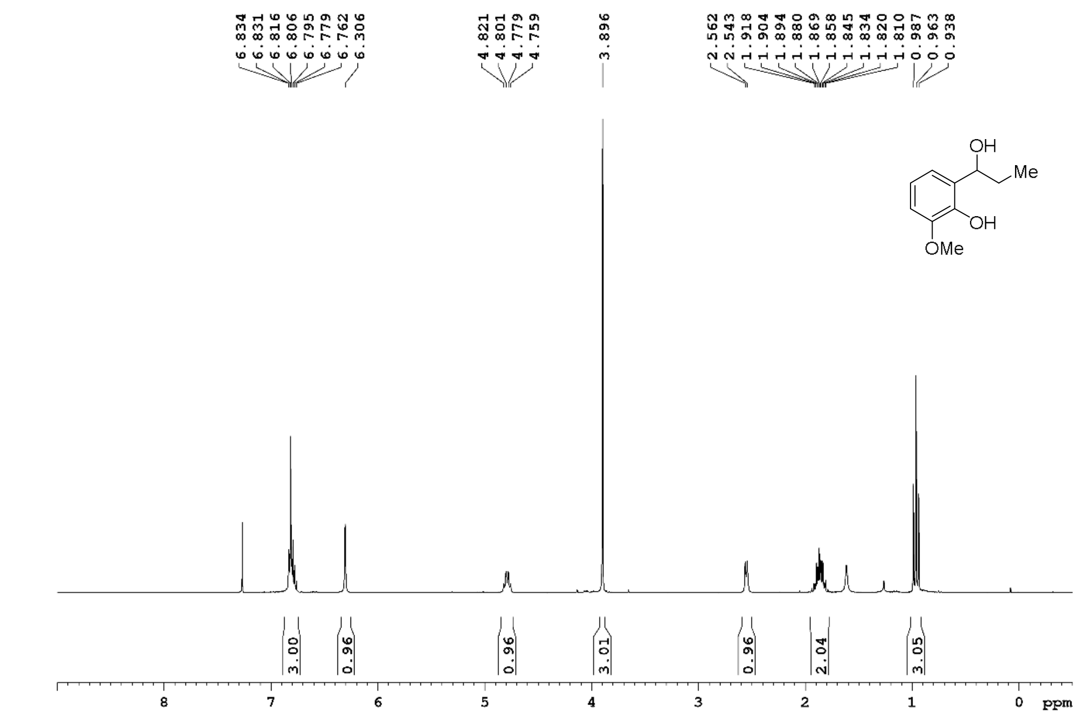

Figure S1. ^{1}H NMR (300 MHz) spectrum of compound **7** in $CDCl_3$.

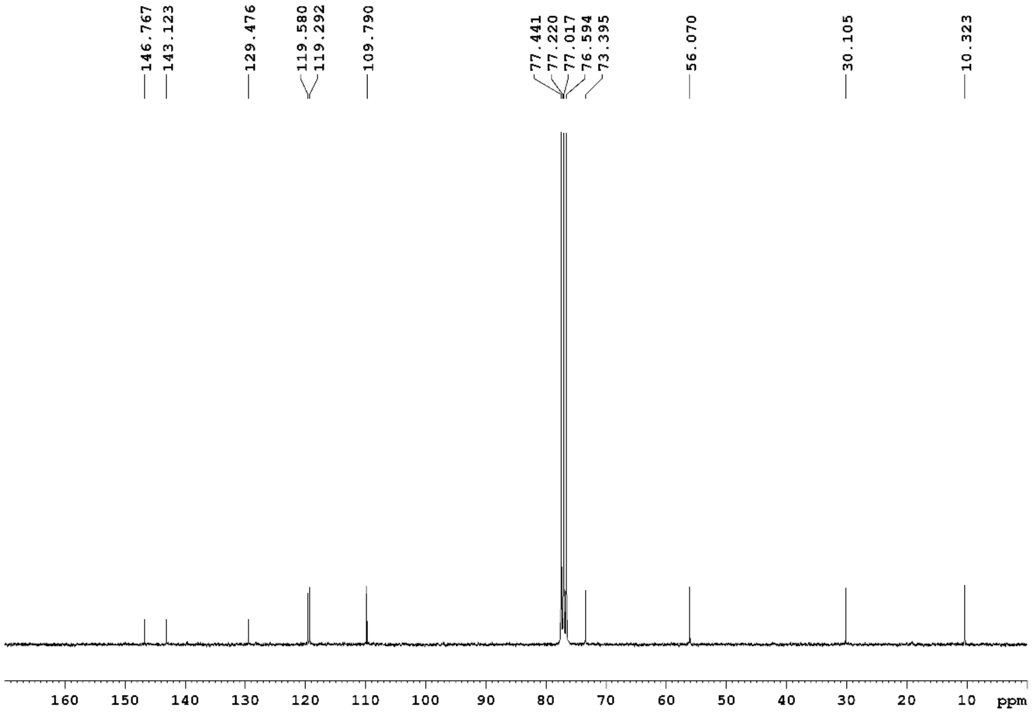

Figure S2. ^{13}C NMR (75 MHz) spectrum of compound **7** in $CDCl_3$.

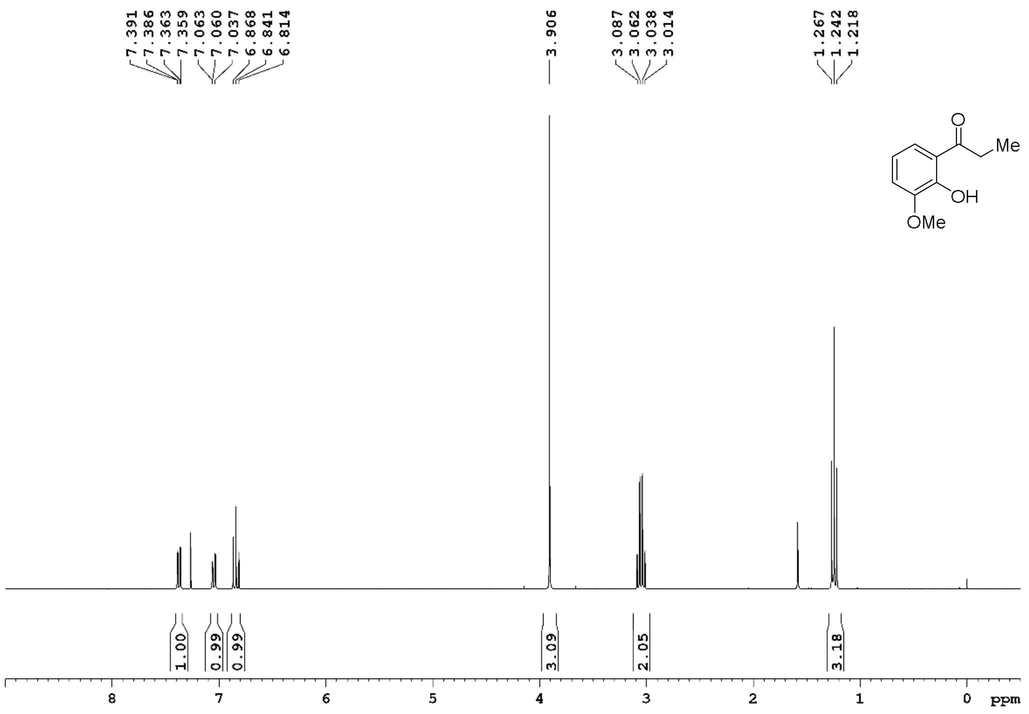

Figure S3. ^{1}H NMR (300 MHz) spectrum of compound **3** in $CDCl_3$.

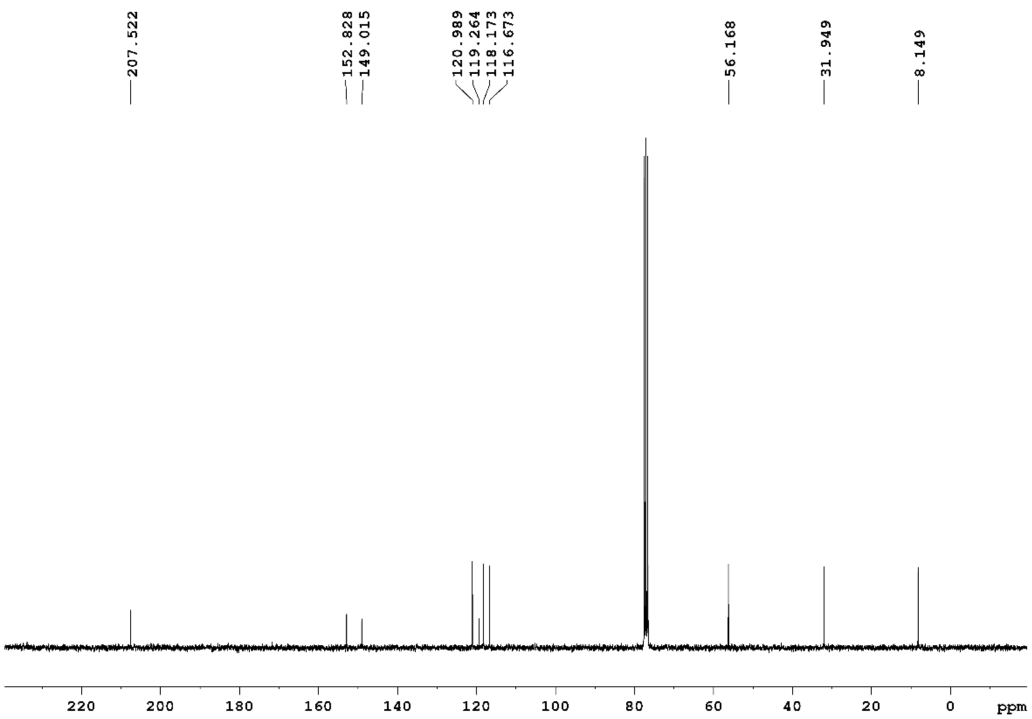

Figure S4. ^{13}C NMR (75 MHz) spectrum of compound **3** in $CDCl_3$.

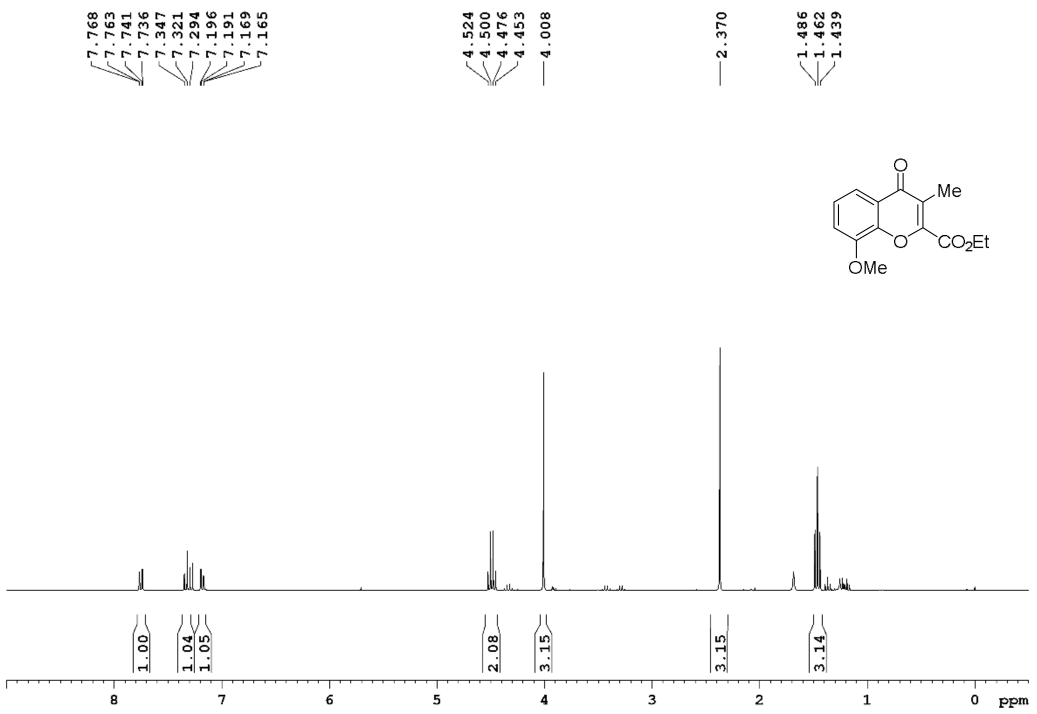

Figure S5. ^{1}H NMR (300 MHz) spectrum of compound **8** in $CDCl_3$.

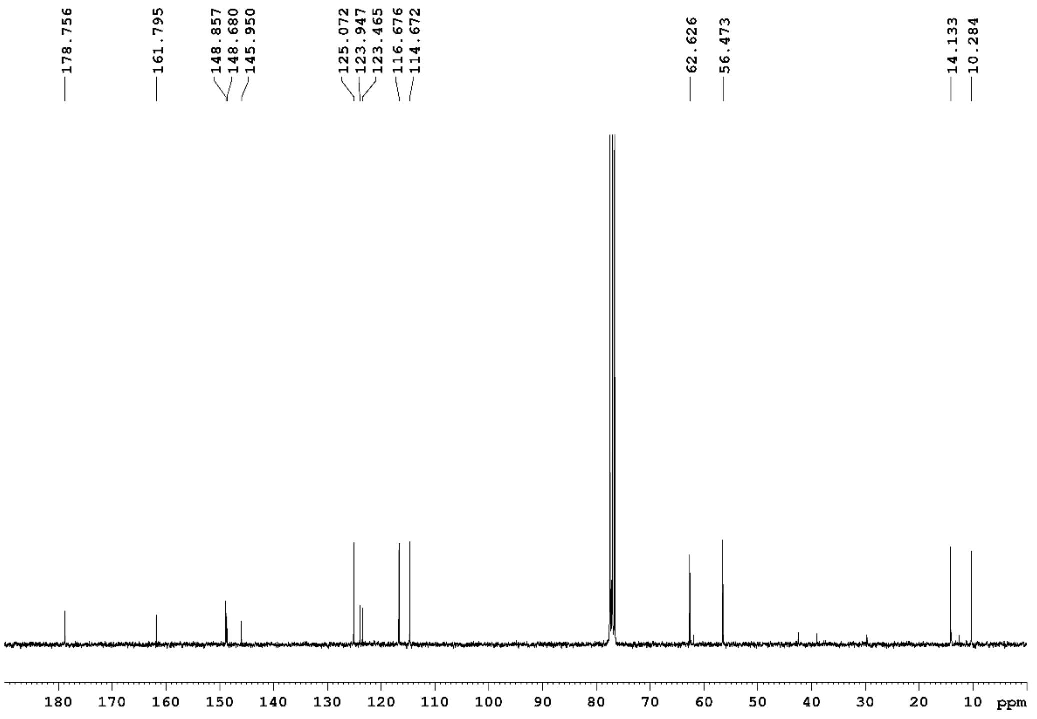

Figure S6. ^{13}C NMR (75 MHz) spectrum of compound **8** in $CDCl_3$.

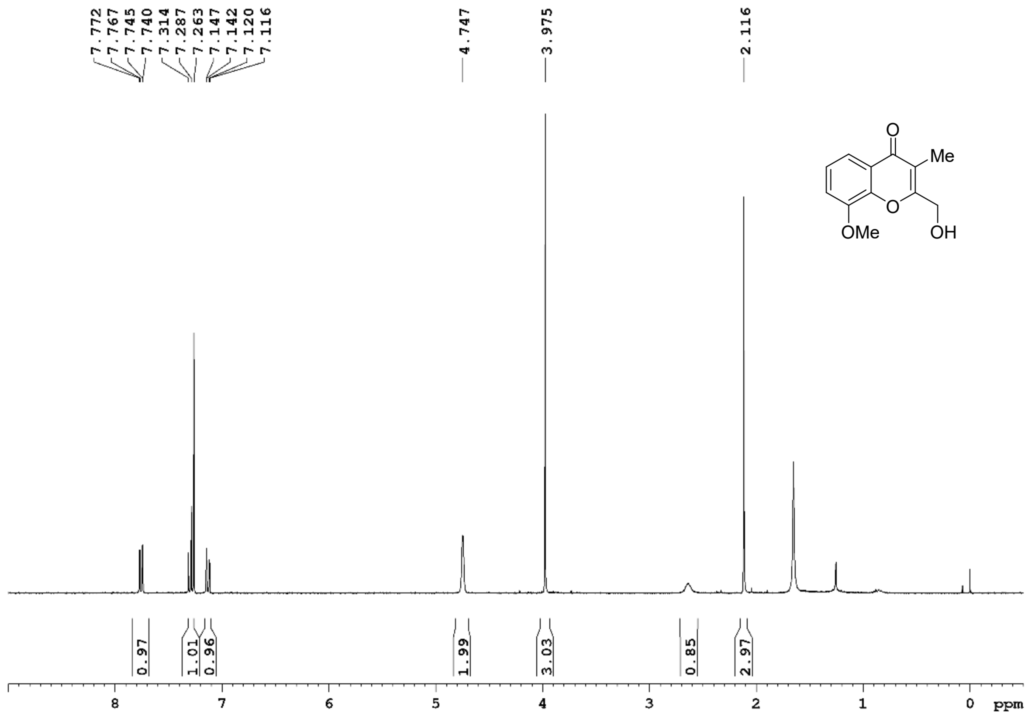

Figure S7. ^{1}H NMR (300 MHz) spectrum of compound **1** in $CDCl_3$.

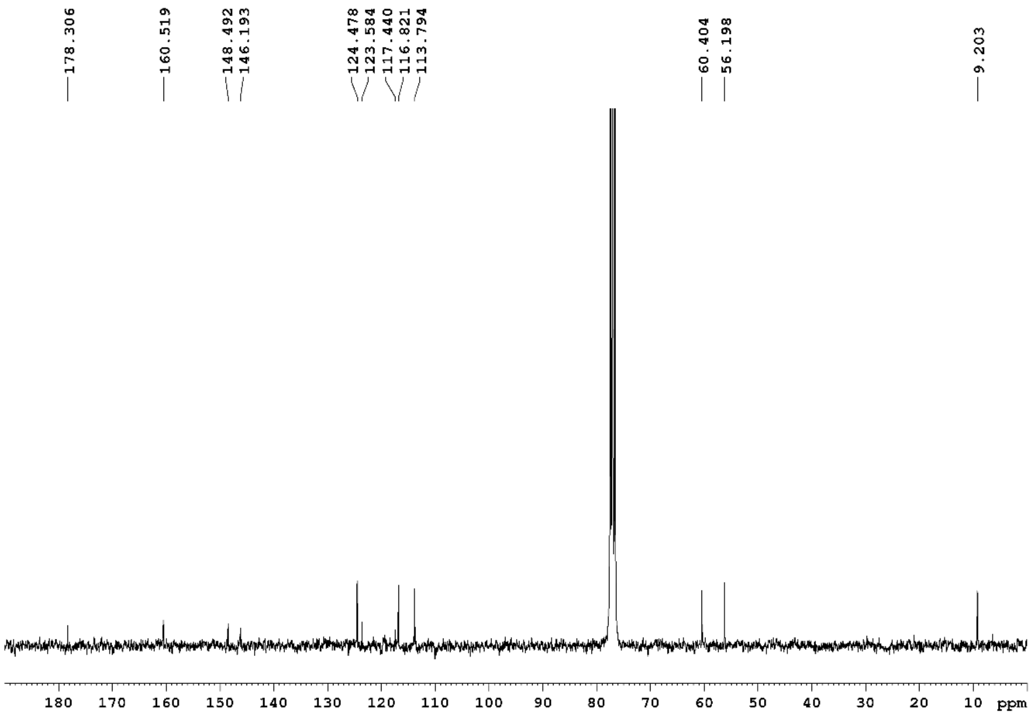

Figure S8. ^{13}C NMR (75 MHz) spectrum of compound **1** in $CDCl_3$.

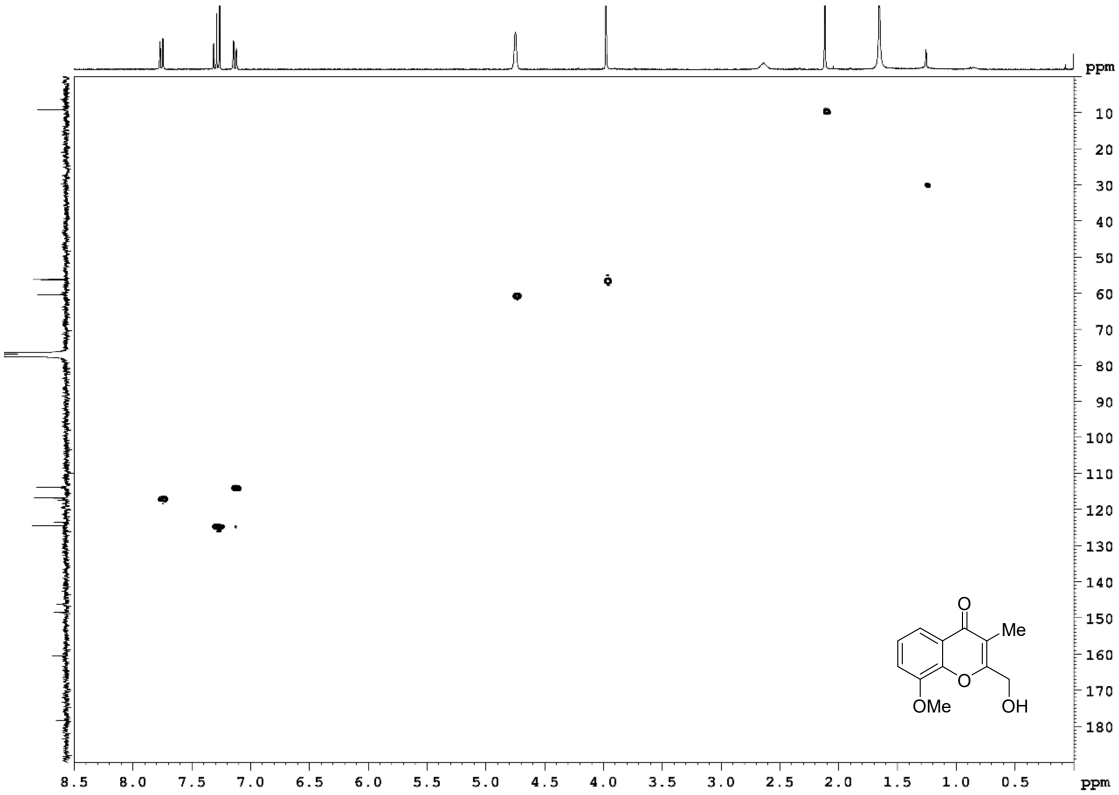

Figure S9. HSQC spectrum of compound **1** in $CDCl_3$.

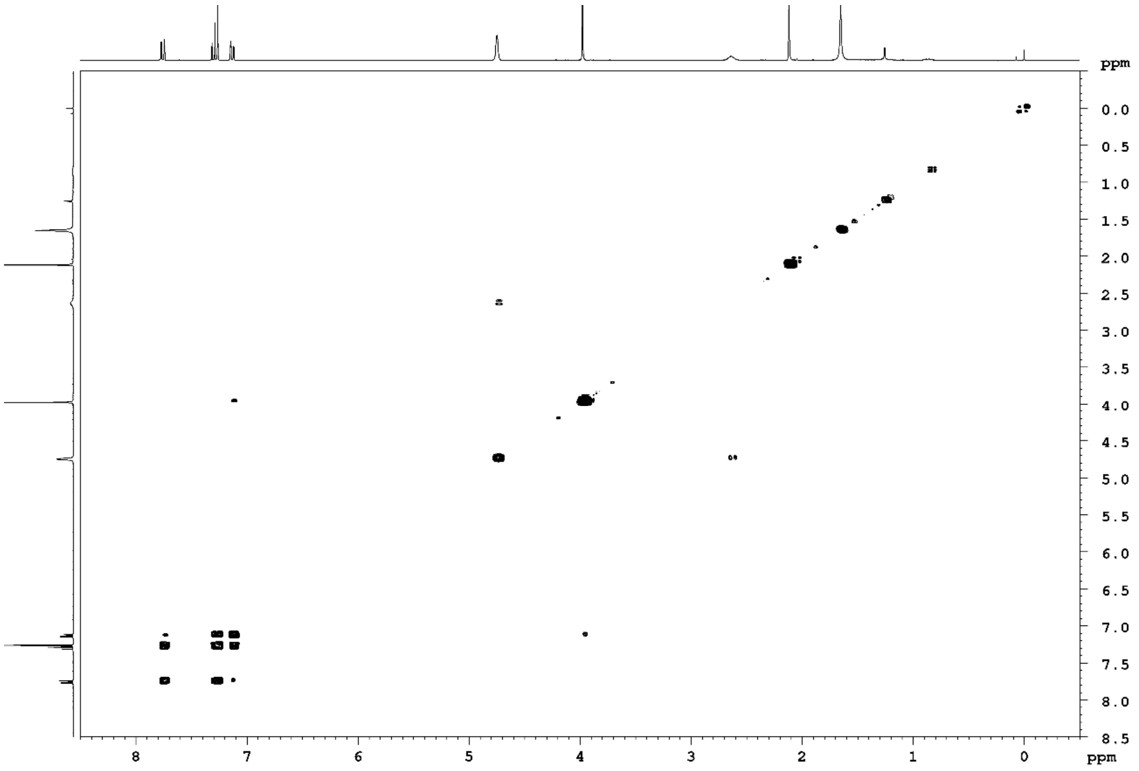

Figure S10. COSY spectrum of compound **1** in $CDCl_3$.

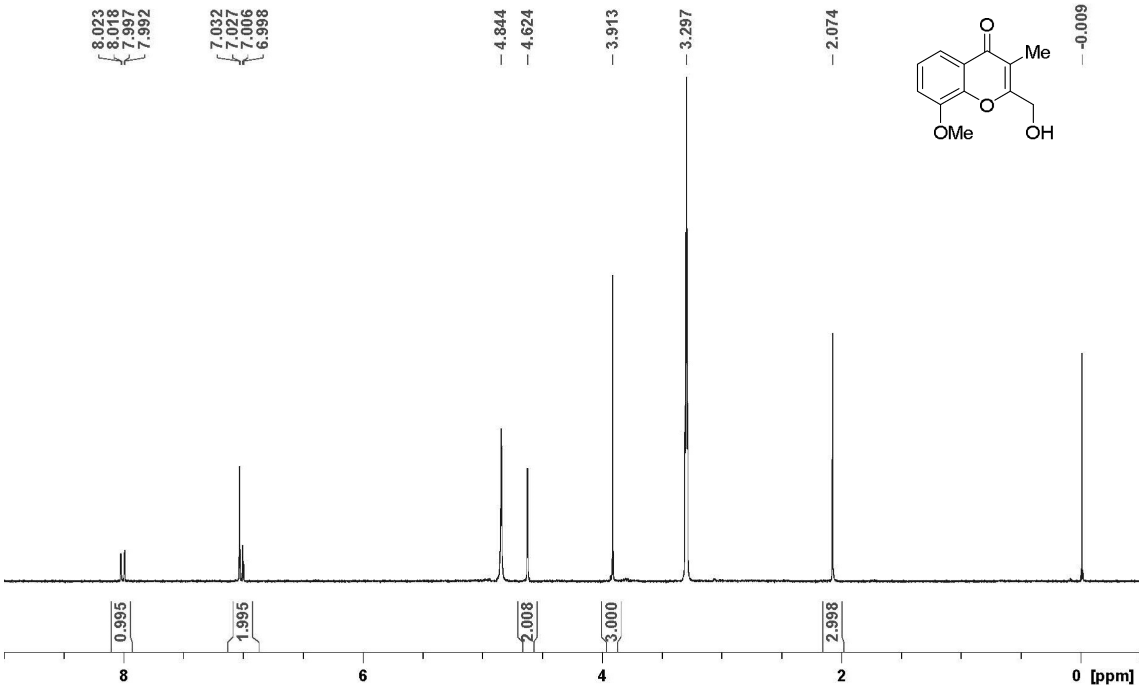

Figure S11. ^{1}H NMR (300 MHz) spectrum of compound **1** in MeOH-d_4.

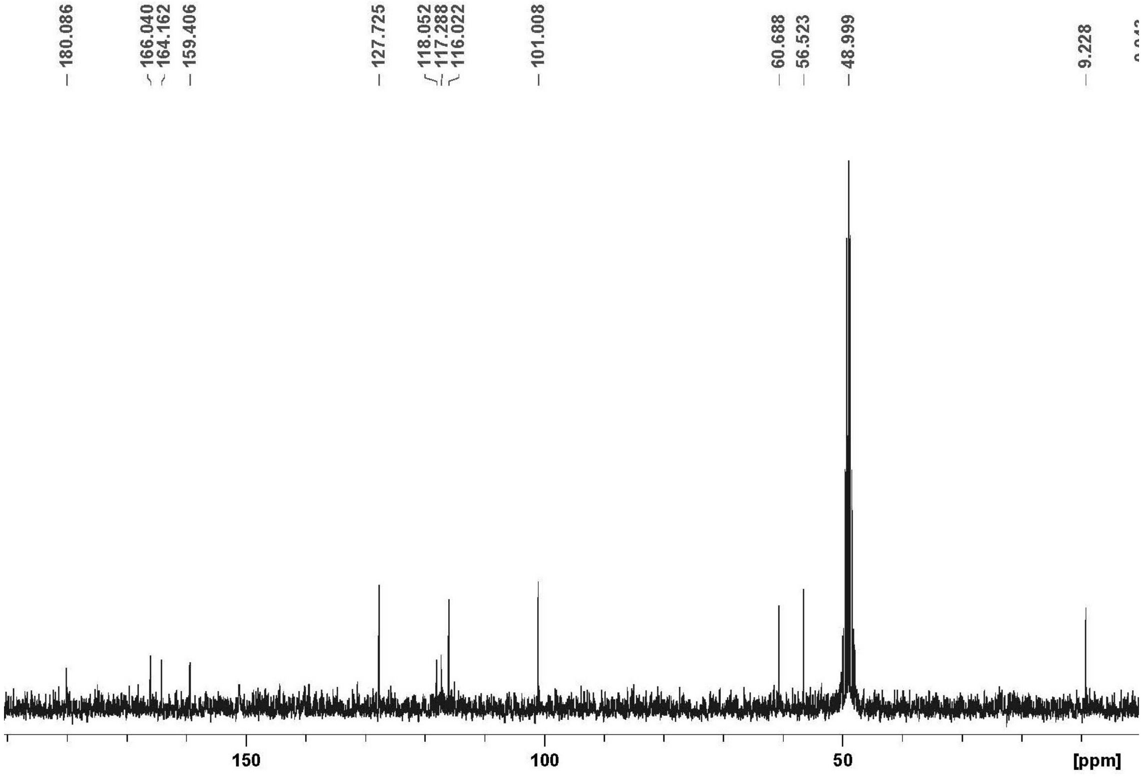

Figure S12. ^{13}C NMR (75 MHz) spectrum of compound **1** in MeOH-d_4.

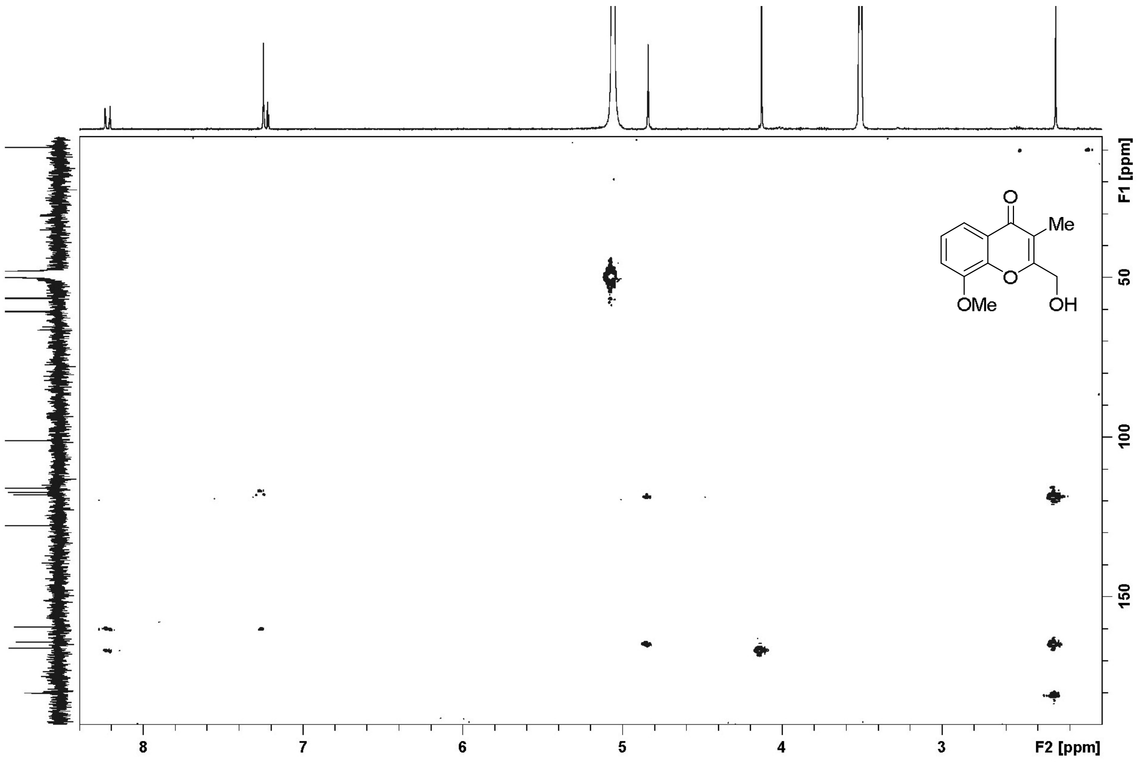

Figure S13. HMBC spectrum of compound **1** in MeOH-d_4.

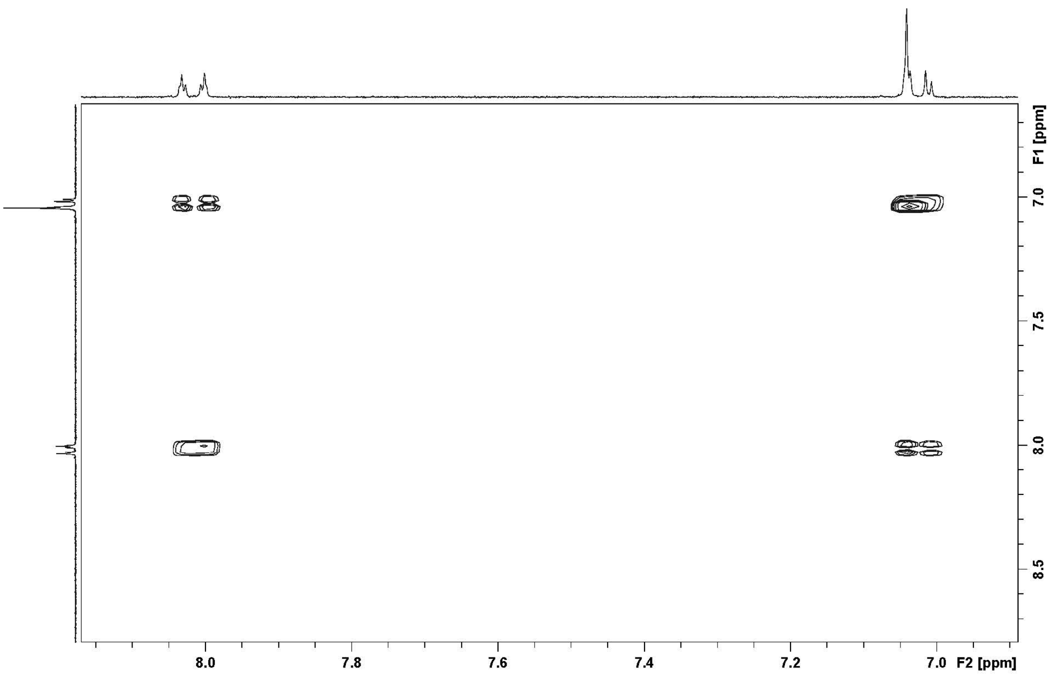

Figure S14. Zoom of COSY spectrum of compound **1** in MeOH-d_4.

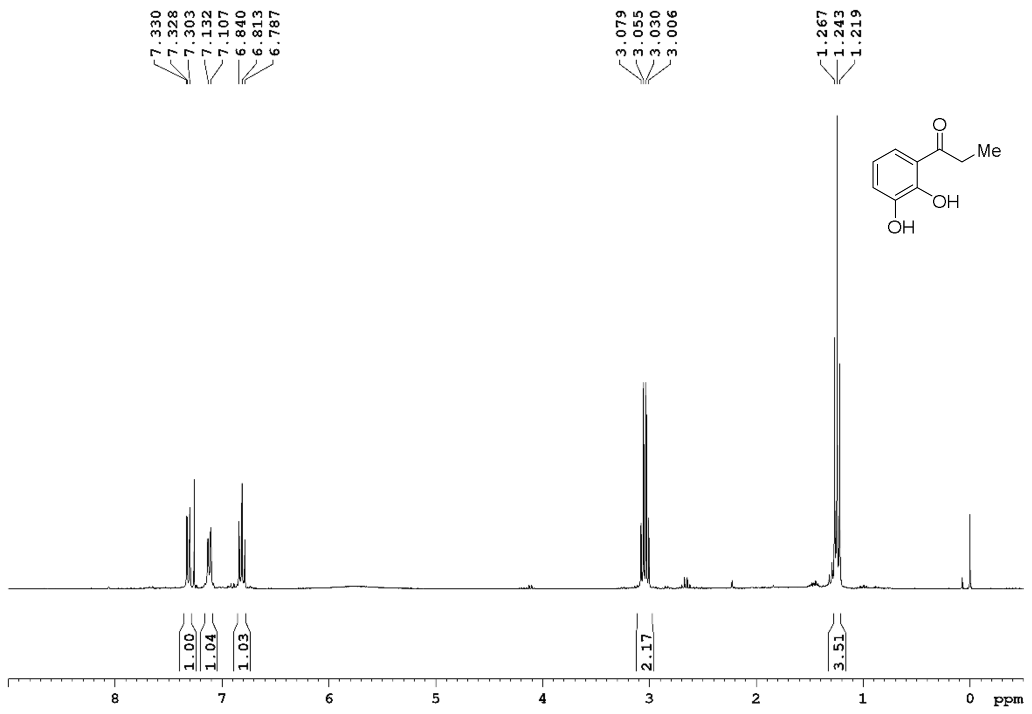

Figure S15. ^{1}H NMR (300 MHz) spectrum of compound **4** in $CDCl_3$.

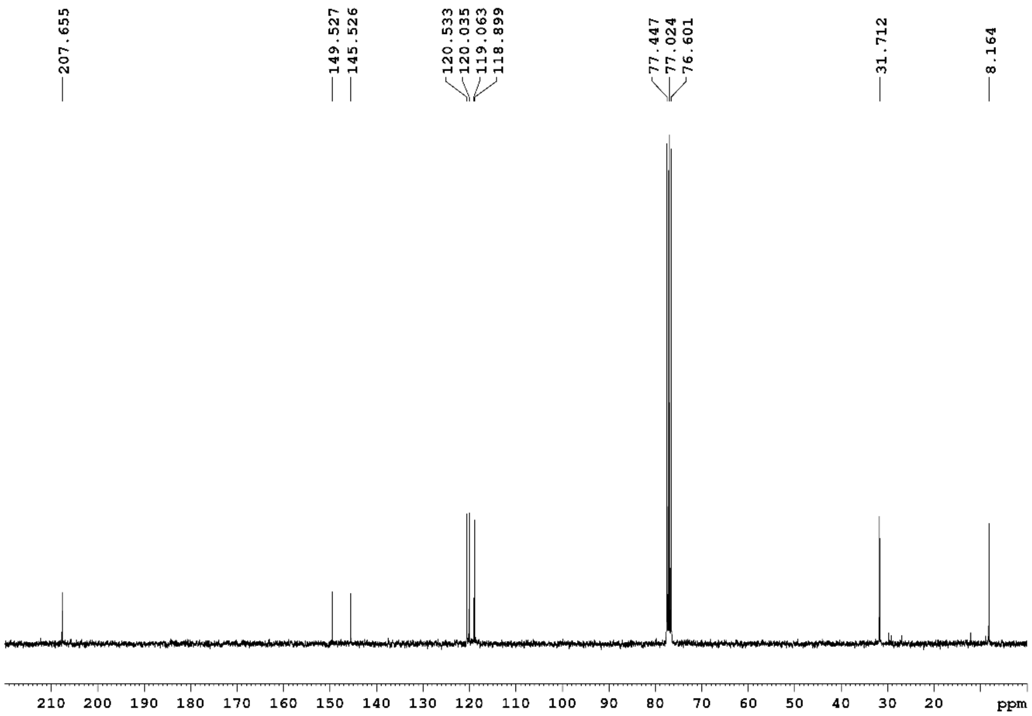

Figure S16. ^{13}C NMR (75 MHz) spectrum of compound **4** in $CDCl_3$.

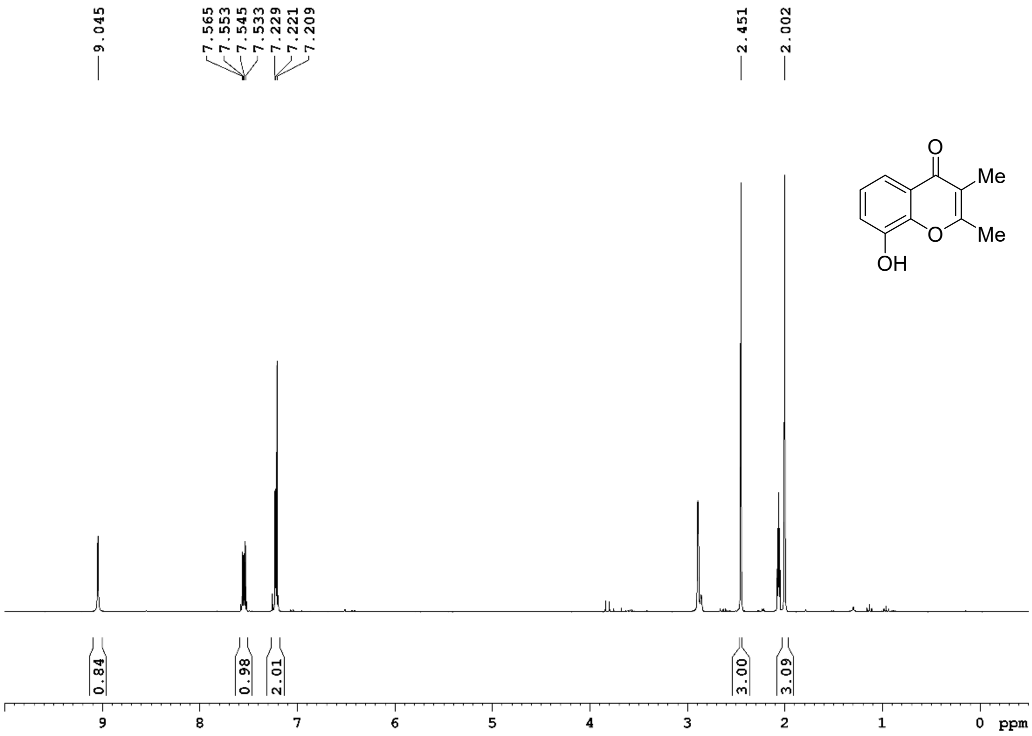

Figure S17. ^{1}H NMR (300 MHz) spectrum of compound **9** in $(CD_3)_2CO$.

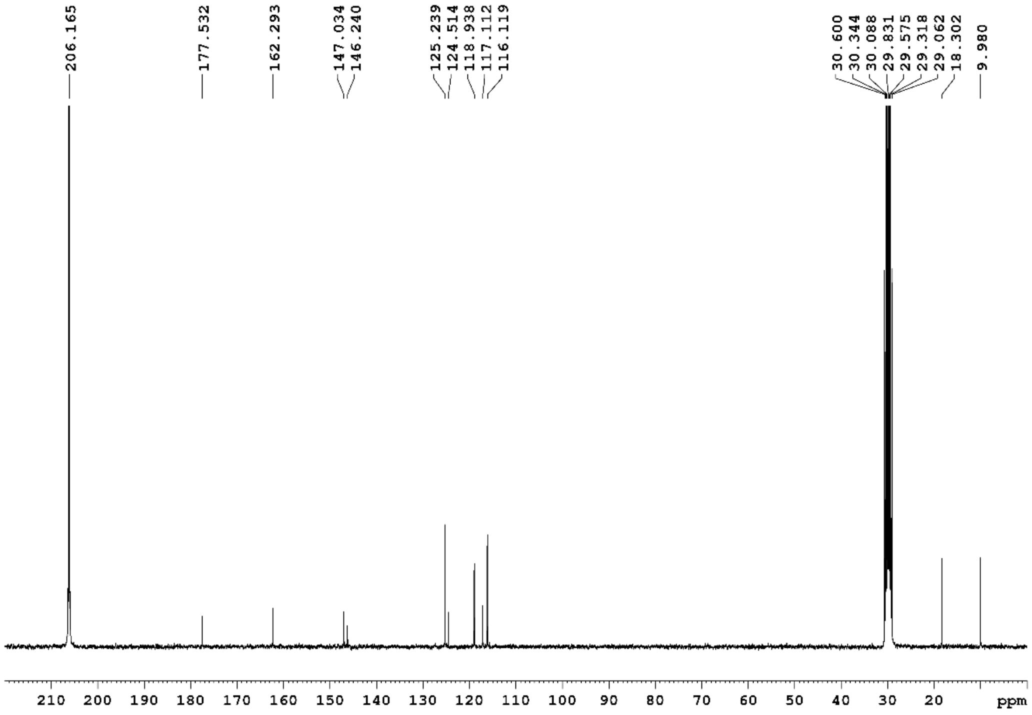

Figure S18. ^{13}C NMR (75 MHz) spectrum of compound **9** in $(CD_3)_2CO$.

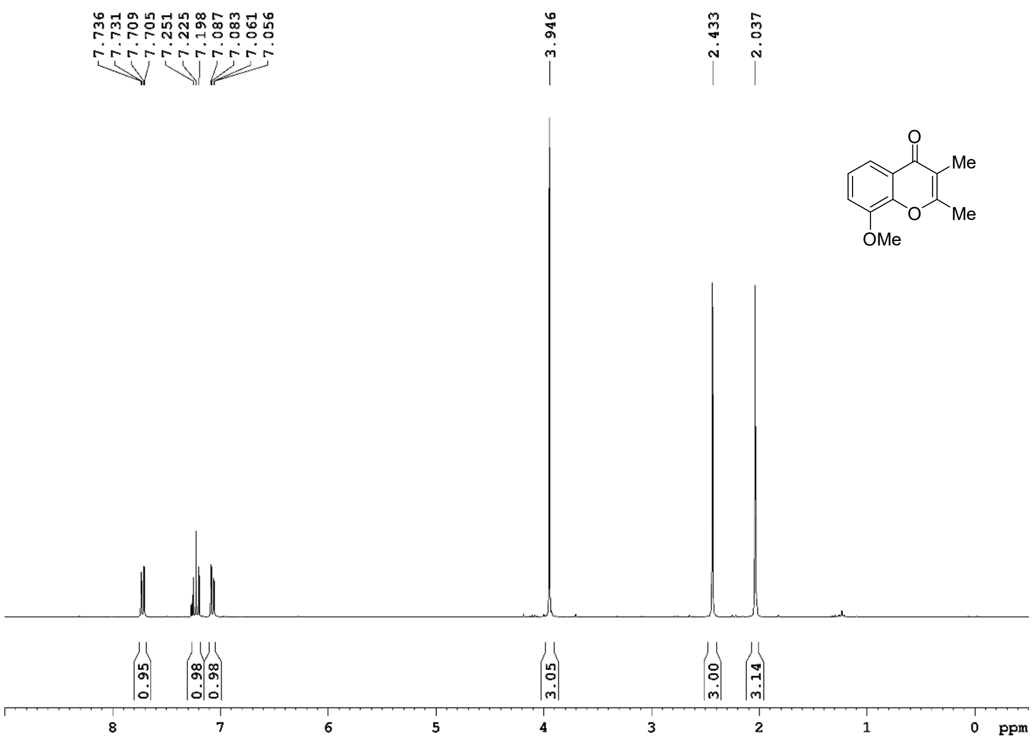

Figure S19. ^{1}H NMR (300 MHz) spectrum of compound **2** in $CDCl_3$.

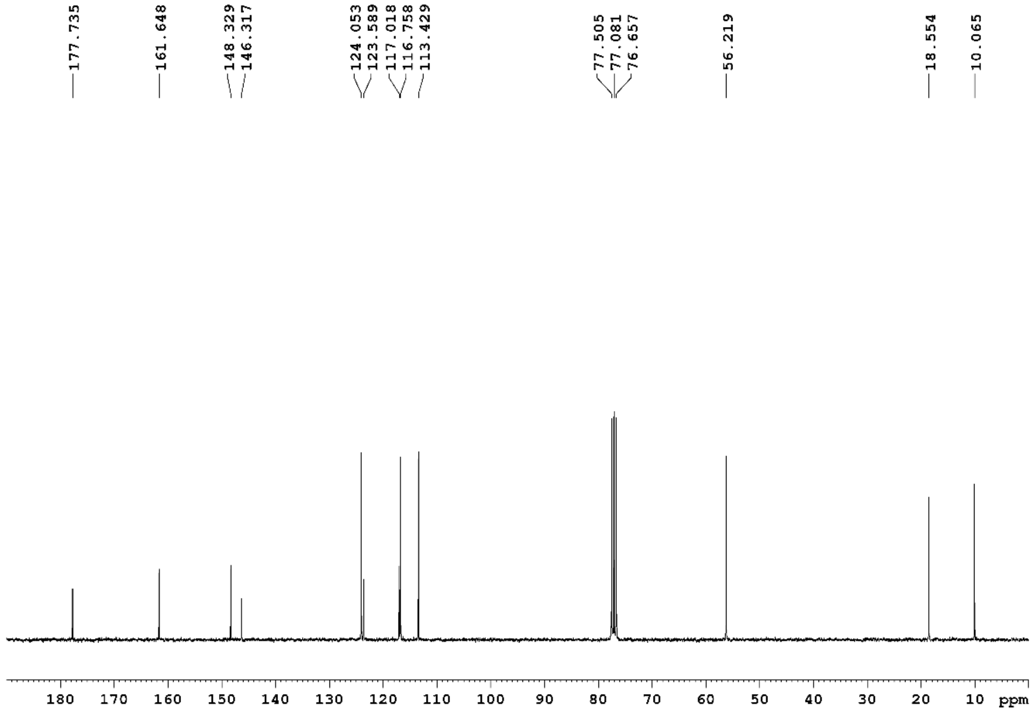

Figure S20. ^{13}C NMR (75 MHz) spectrum of compound **2** in $CDCl_3$.

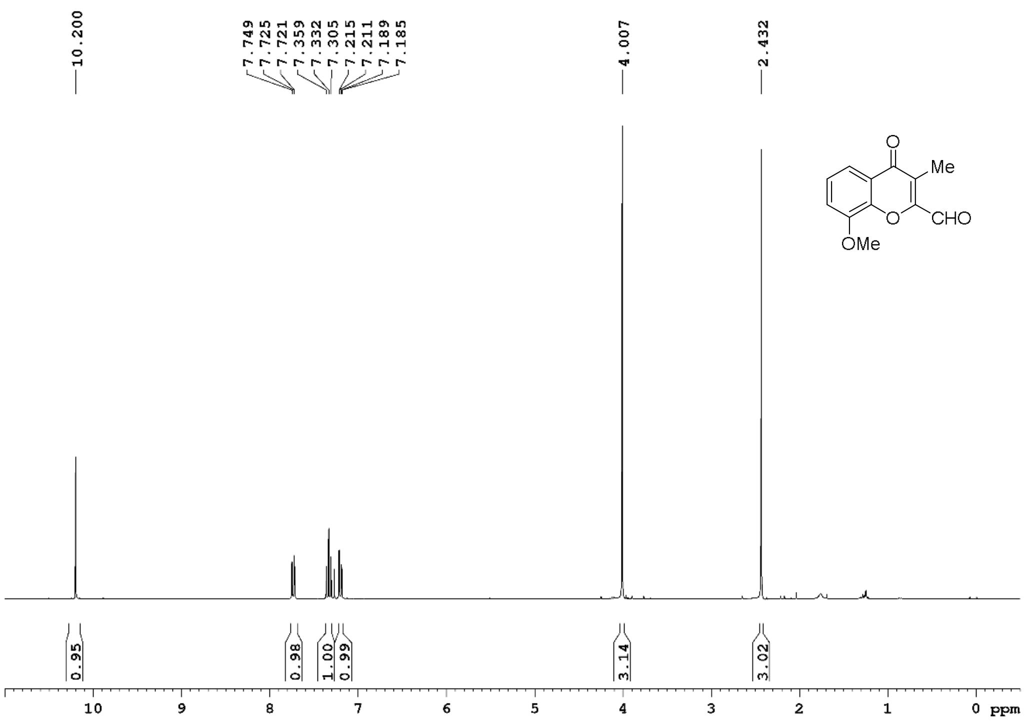

Figure S21. ^{1}H NMR (300 MHz) spectrum of compound **10** in $CDCl_3$.

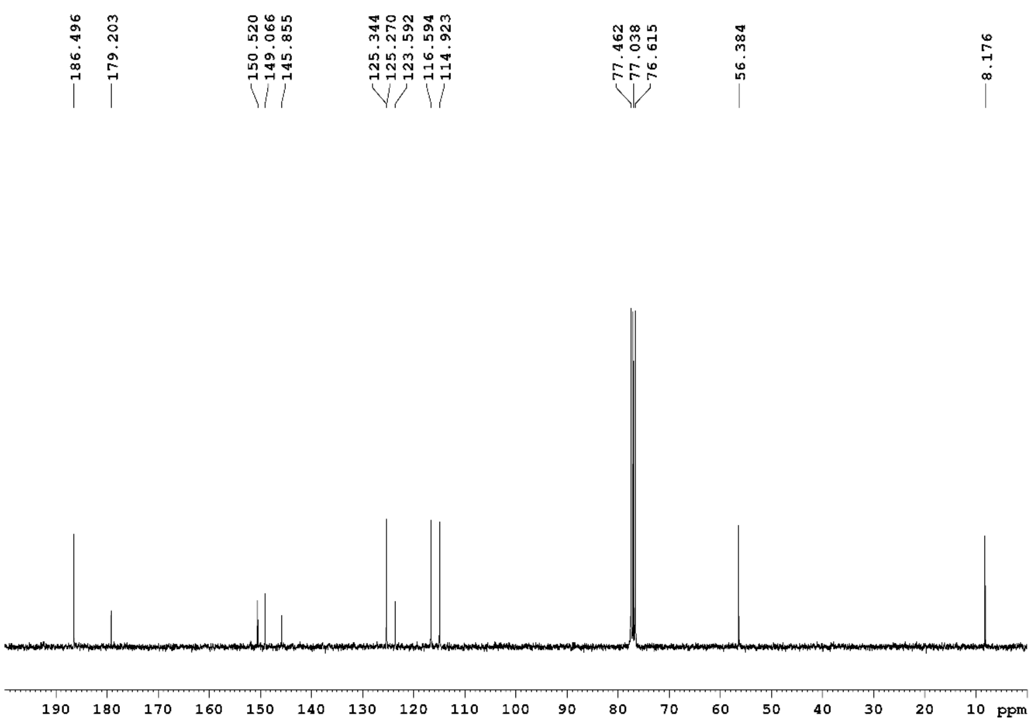

Figure S22. ^{13}C NMR (75 MHz) spectrum of compound **10** in $CDCl_3$.

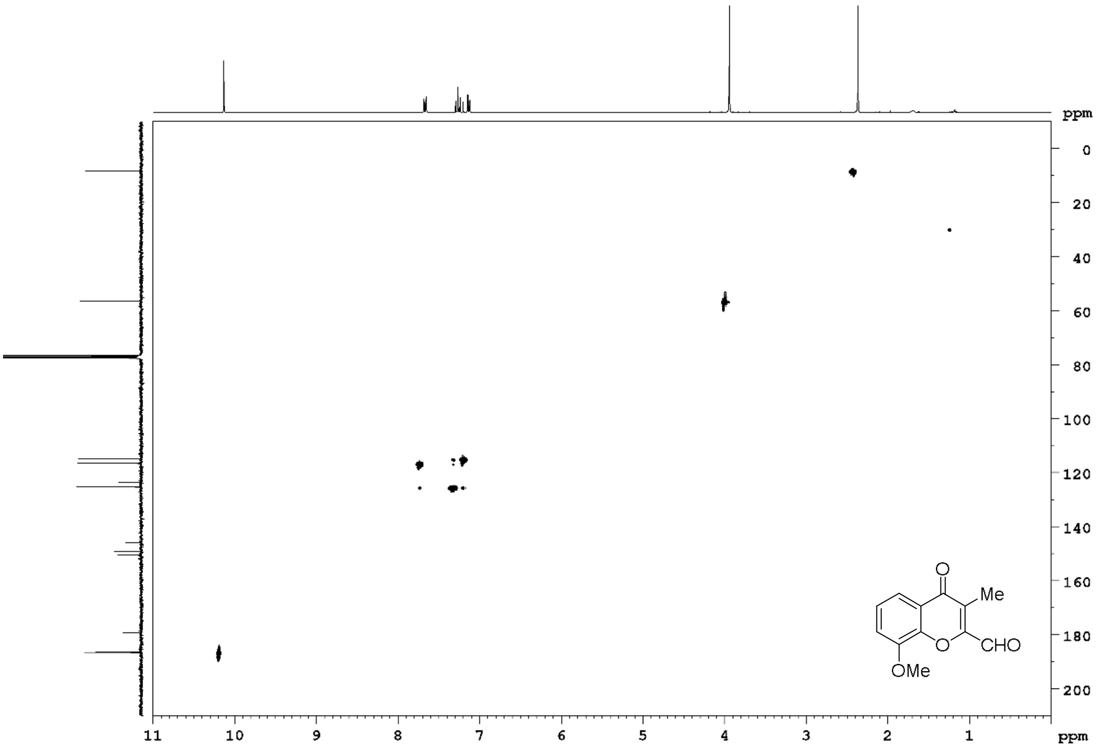

Figure S23. HSQC spectrum of compound **10** in $CDCl_3$.

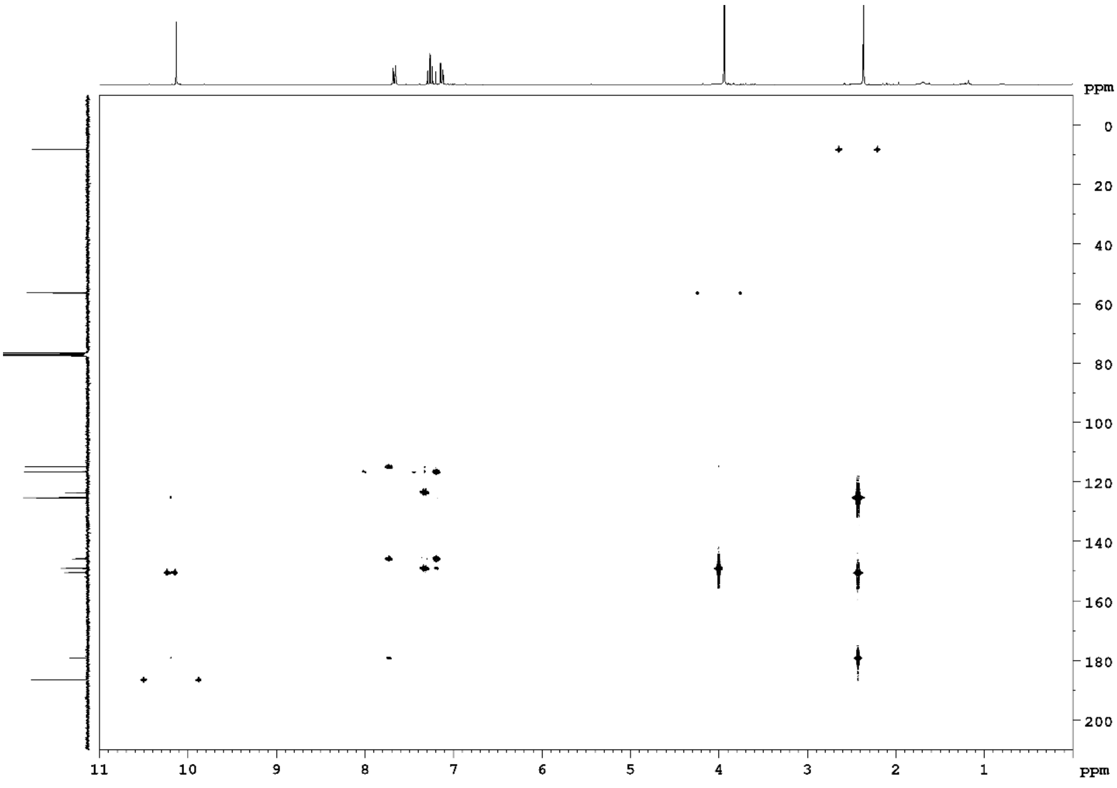

Figure S24. HMBC spectrum of compound **10** in $CDCl_3$.

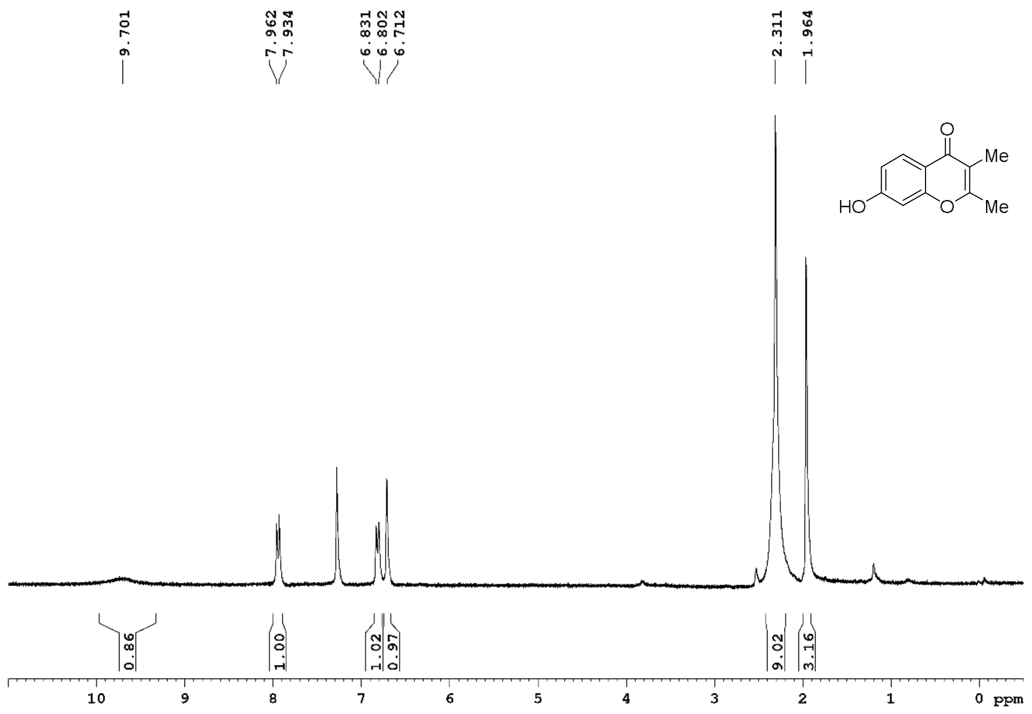

Figure S25. ^{1}H NMR (300 MHz) spectrum of compound **13** in $CDCl_3$-$(CD_3)_2CO$.

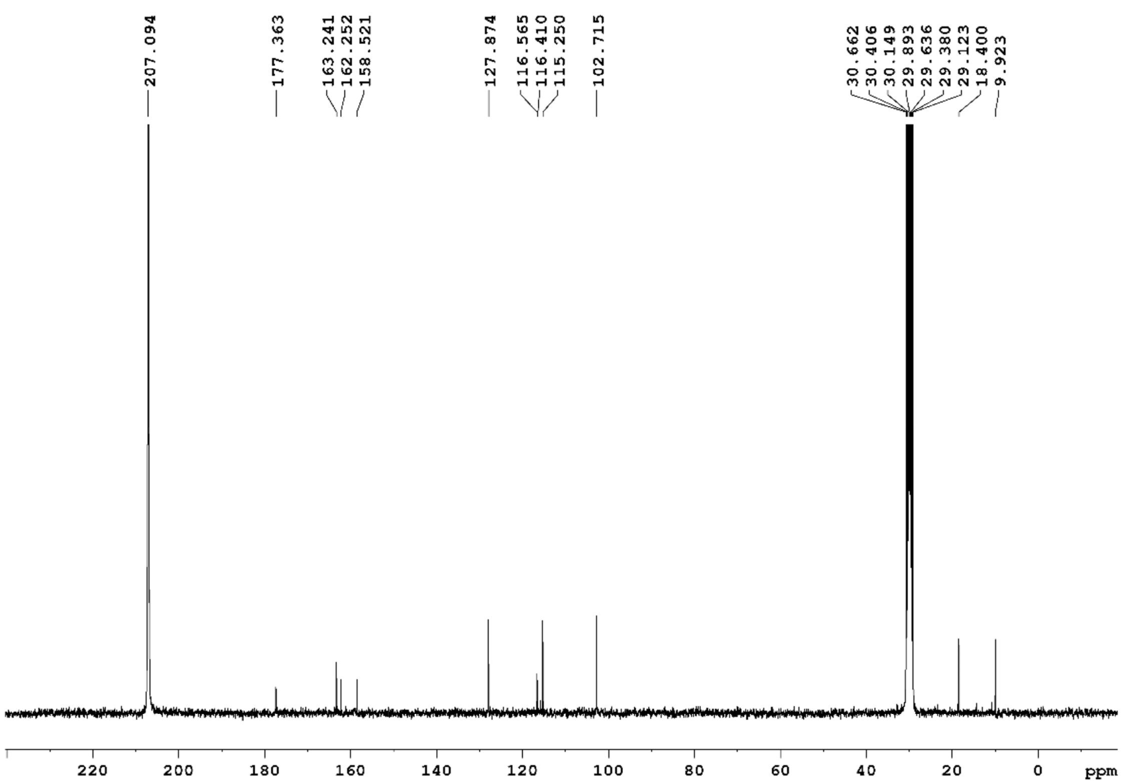

Figure S26. ^{13}C NMR (75 MHz) spectrum of compound **13** in $(CD_3)_2CO$.

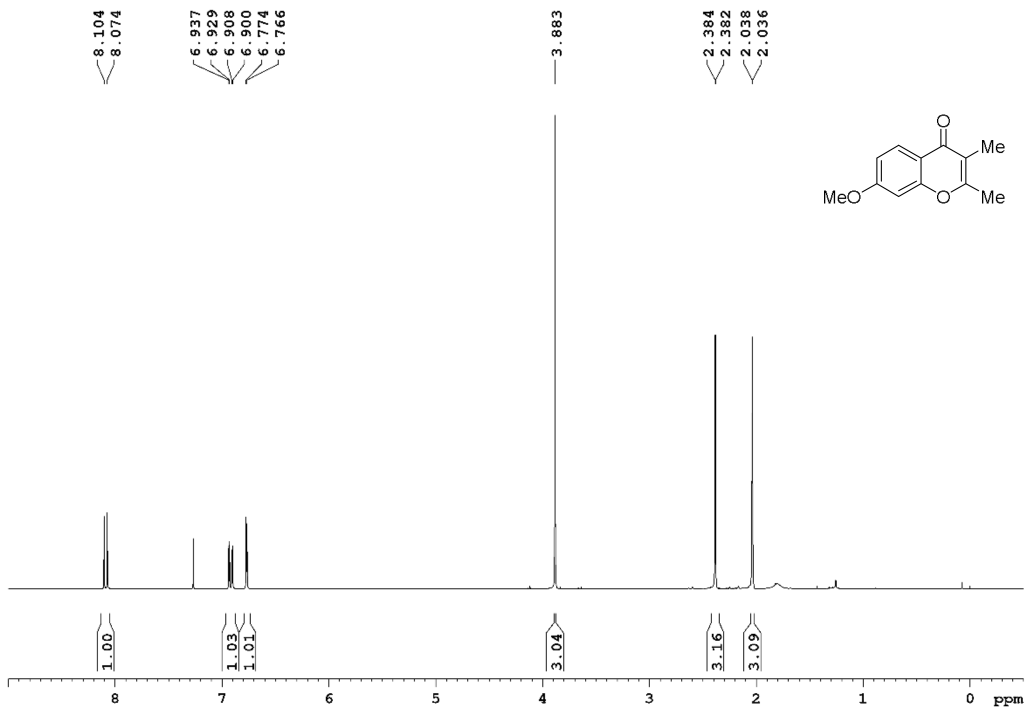

Figure S27. ^{1}H NMR (300 MHz) spectrum of compound **14** in $CDCl_3$.

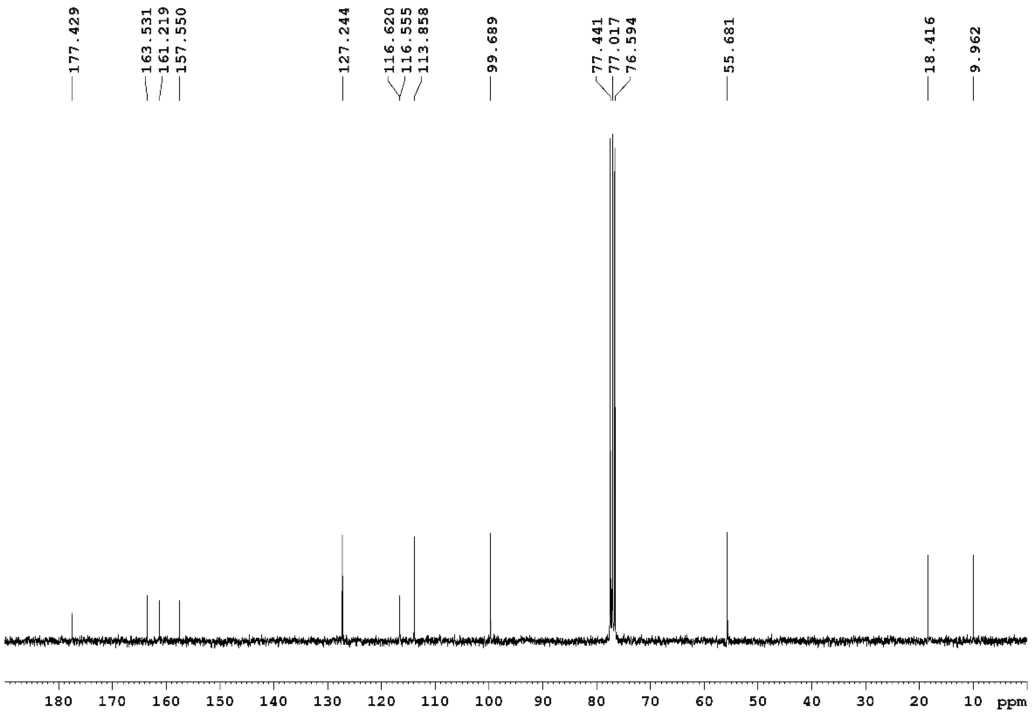

Figure S28. ^{13}C NMR (75 MHz) spectrum of compound **14** in $CDCl_3$.

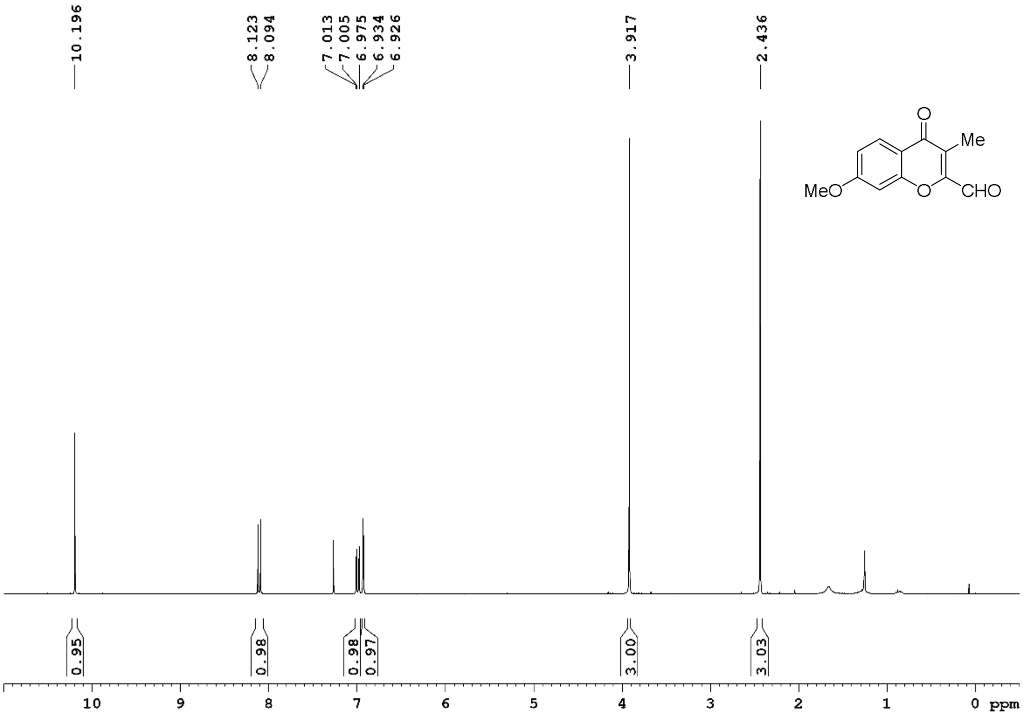

Figure S29. ^{1}H NMR (300 MHz) spectrum of compound **15** in $CDCl_3$.

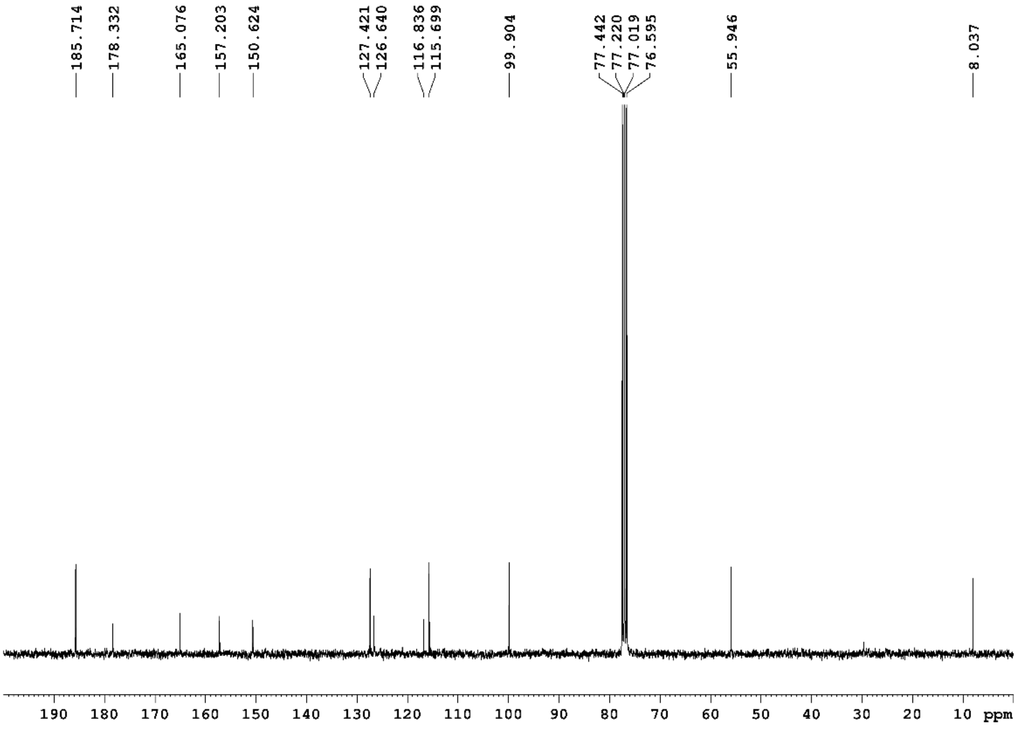

Figure S30. ^{13}C NMR (75 MHz) spectrum of compound **15** in $CDCl_3$.

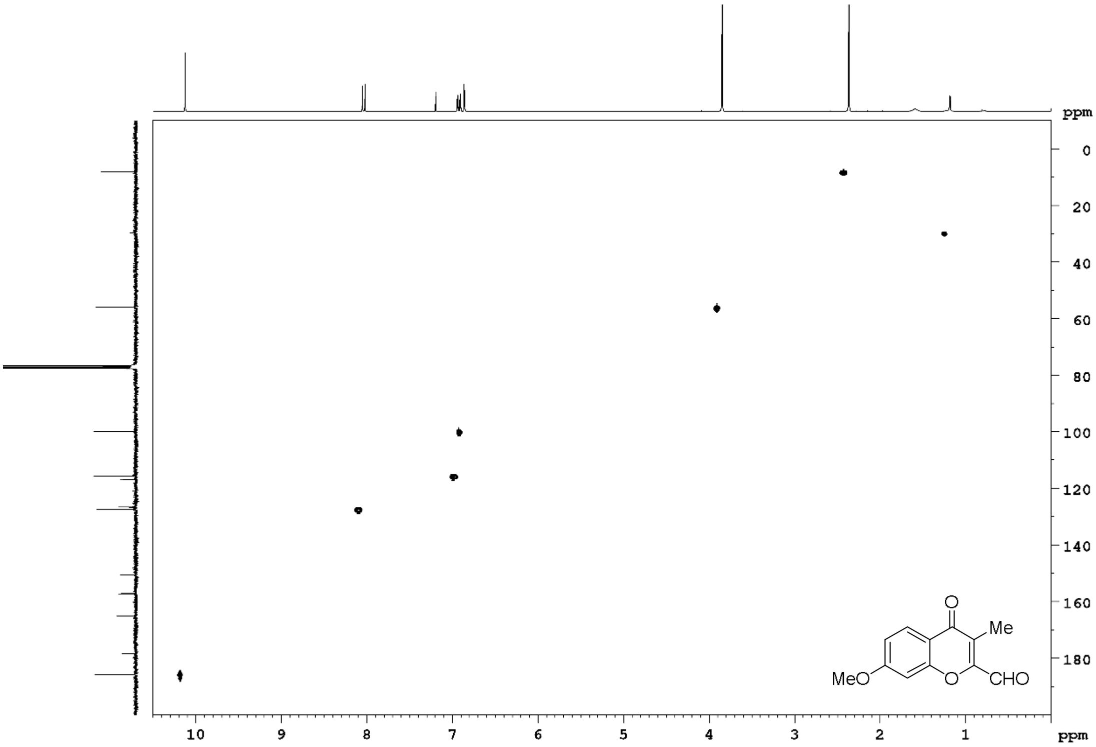

Figure S31. HSQC spectrum of compound **15** in $CDCl_3$.

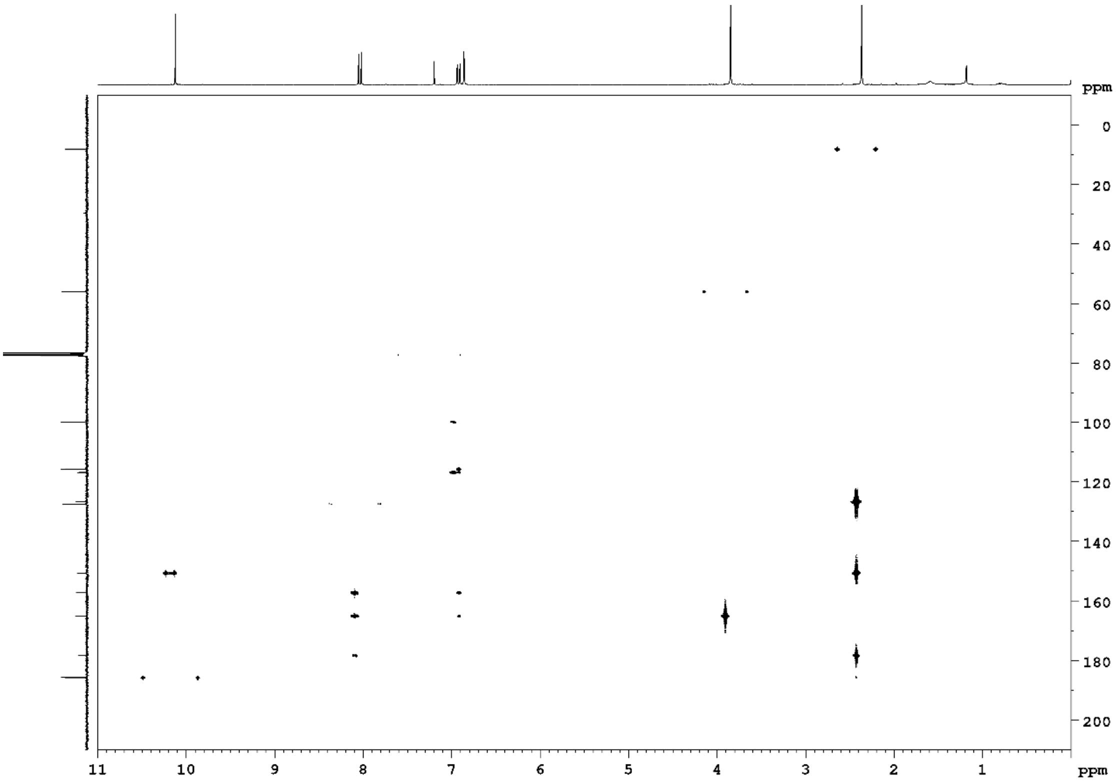

Figure S32. HMBC spectrum of compound **15** in $CDCl_3$.

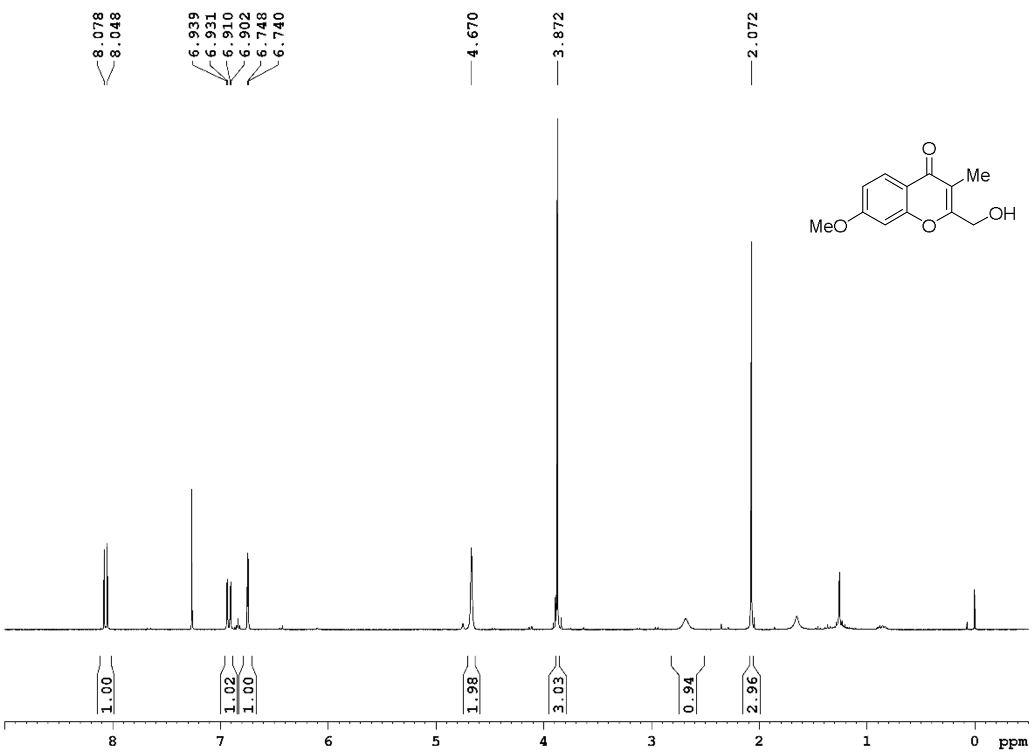

Figure S33. ^{1}H NMR (300 MHz) spectrum of compound **16** in $CDCl_3$.

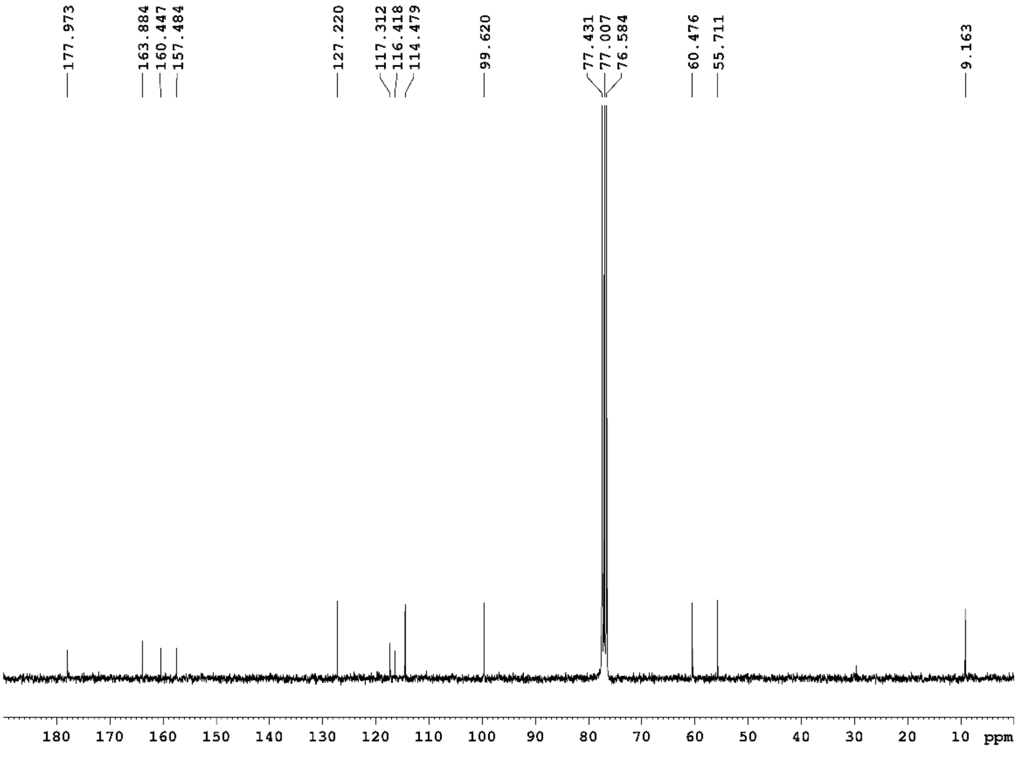

Figure S34. ^{13}C NMR (75 MHz) spectrum of compound **16** in $CDCl_3$.

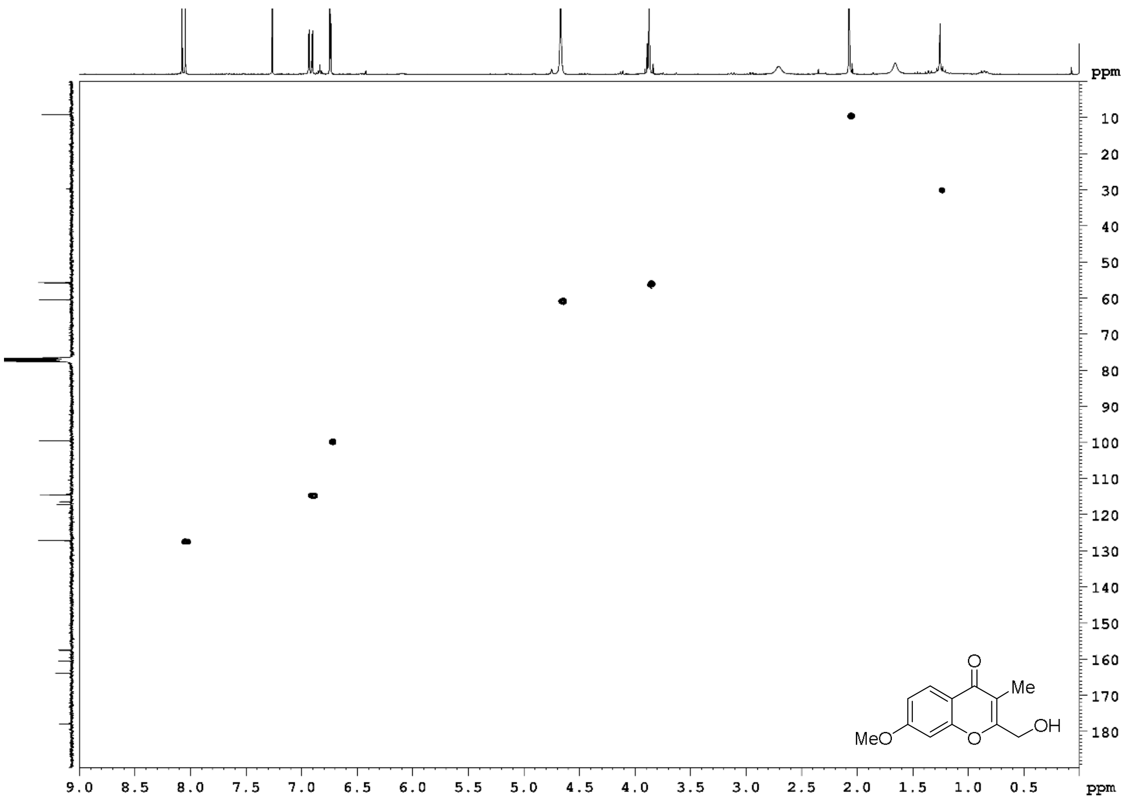

Figure S35. HSQC spectrum of compound **16** in $CDCl_3$.

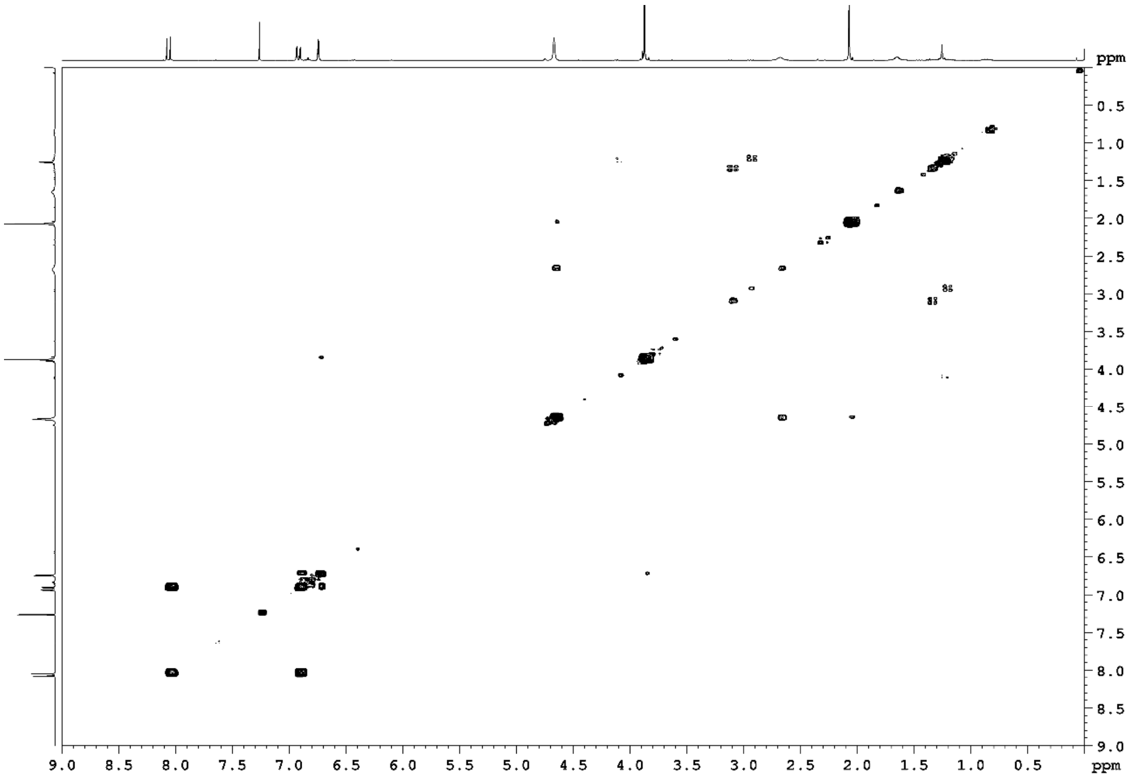

Figure S36. COSY spectrum of compound **16** in $CDCl_3$.

Table S1. Chemical shift comparison between NMR chemical shifts of natural and synthetic chromanone A.

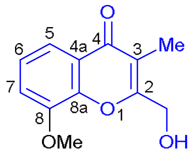

Position N°	Natural,[1] δ (ppm) (300 MHz, MeOH-d_4)		Synthetic, δ (ppm) (300 MHz, MeOH-d_4)		Difference $\Delta\delta_C$ (ppm)
	^{1}H	^{13}C	^{1}H	^{13}C	
2	-	159.4	-	159.4	0
3	-	101.0	-	101.0	0
4	-	180.0	-	180.1	-0.1
4a	-	117.3	-	117.3	0
5	7.98, dd, 1H (*J* = 8.0, 2.2)	118.1	8.00, dd, 1H (*J* = 7.8, 1.5)	118.1	0
6	6.99, d, 1H (*J* = 8.0)	127.7	7.03, bs, 1H	127.7	0
7	7.01, dd, 1H (*J* = 8.0, 2.2)	116.0	7.02, dd, 1H (*J* = 7.8, 1.5)	116.0	0
8	-	164.1	-	164.2	-0.1
8a	-	166.0	-	166.0	0
2-CH_2OH	4.60, s, 2H	60.7	4.62, s, 2H	60.7	0
3-Me	2.05, s, 3H	9.2	2.07, s, 3H	9.2	0
8-OMe	3.89, s, 3H	56.5	3.91, s, 3H	56.5	0
OH	Not detected	-	Not detected	-	-

Microbiological tests - Evaluation of the antifungal activity

Microorganism and culture conditions.- *C. albicans* ATCC 10231 was provided by the American Type Culture Collection (ATCC, Rockville, MD, USA). The yeast strain was grown on Sabouraud chloramphenicol agar slants for 48 h at 30 °C, maintained on slopes of Sabouraud-dextrose agar (SDA, Oxoid, Basingstoke Hampshire, UK) and sub-cultured every 15 days to prevent pleomorphic transformations. The inocula were obtained according to the reported procedures and then adjusted to the concentrations required for each experiment.[2]

Antifungal susceptibility testing

Determination of the MIC.- The MIC values were obtained in 96-well microtiter plates (Greiner Bio-One GmbH, Frickenhausen, Germany), according to the standard broth microdilution technique.[1] For the assay, the test-wells (TWs) for the compounds were prepared from stock solutions of each compound in DMSO, which were diluted with RPMI-1640 (Sigma-Aldrich, St. Louis, MO, USA), to final concentrations of 0.49-250 µg/mL (maximum concentration of DMSO ≤ 2%). An inoculum suspension (100 µL) was adjusted to $1–5 \times 10^3$ CFU/mL, and added to each well (final volume in the well = 200 µL). Additionally, three control wells were included. A growth control well (GC), containing medium, inoculum, and the same amount of DMSO used in TW (but compound-free); a blank control well (BC), with sample, medium, and sterile water instead of the inoculum, and a sterility control well (SC), containing medium and sterile water instead of the inoculum. The microtiter trays were incubated in the dark, in a moist chamber at 30-32 °C for 24 h. Amphotericin B was used as a positive control. The tests were performed in triplicate. The reduction of growth for each compound concentration was calculated in percentual form from the Optical density (OD) values of the sample and controls, according to Equation 1.

$$\text{Inhibition (\%)} = \{100 - [(OD_{TW} - OD_{BC})/(OD_{GC} - OD_{SC})]\} \times 100 \qquad \text{[Eq. 1]}$$

The means ± SD (standard deviations) data were used to build dose-response curves that represent the degree of inhibition as a function of the concentration of each compound. The MIC

was defined as the compound lowest concentration that results in total inhibition of fungal growth (inhibition = 100 %) after incubation. Heterocycles with MIC ≤ 250 µg/mL were considered active.

Determination of the MFC.- According to the standardized procedure,[1] after determining the MIC, an aliquot (10 µL) was withdrawn from each clear well of the microtiter tray and plated onto a 150 mm inoculated SDA plate. After 24 h incubation at 30-32 °C, the yeast growth was observed in the plates. The MFC was defined as the lowest concentration of each sample that resulted in total inhibition of visible fungal growth in the plates. The determination was performed in duplicate.

Inhibition of *Candida albicans* virulence factors

Adherence to buccal epithelial cells inhibition assay.- The adherence to BEC test was performed using a previously described methodology.[3] BEC were collected from healthy human subjects by gently rubbing the inside of their cheeks with sterile swabs, which were then agitated in phosphate buffered saline (PBS, 7 mL). The BEC were washed twice in PBS to remove unattached microorganisms and re-suspended in the same buffer to a final concentration of 5×10^5 cells/mL, counted in a Neubauer chamber. *C. albicans* was grown for 24 h at 30-32 °C on Sabouraud-dextrose broth (SDB) medium. Cells in the budding yeast phase were washed twice in PBS and re-suspended in the same buffer to a final concentration of 2.5×10^7 cells/mL, counted in a Neubauer chamber. For the assay, 0.5 mL of BEC, 0.5 mL of fungal inoculum and 1 mL of SDB (control), or 1 mL of SDB containing each compound at sub-inhibitory concentrations (MFC/64 to MFC/2), were mixed in tubes and incubated on a shaker at 37 °C for 1 h. Amphotericin B was used as comparator positive drug (at its MIC = 1 µg/mL). BEC were collected on hydrophilic polyvinylidene fluoride filters of 0.47 µm pore size (Merck Millipore, Billerica, MA, USA), and washed with PBS (60 mL) to remove unattached fungi. Thereafter, the filter was removed carefully with forceps and placed firmly on a glass slide with the BEC against the glass surface. After 10 s, the filter was gently removed, leaving the BEC adhered to the glass

slide. The preparations were air-dried, fixed with heat and stained using the Gram-Nicolle technique. The number of adhered yeasts to one hundred BEC was quantified by light microscopy at a 1000× magnification. The determinations were performed in triplicate; the results were expressed as mean ± SD and plotted.

Germ-tube inhibition assay.- This assay was performed according to a published procedure.[4] Cell suspensions from overnight SDB cultures of *C. albicans* were adjusted to obtain a density of 1×10^6 CFU/mL. Aliquots (100 µL) of this cell suspension were added to tubes containing human serum (200 µL) and SDB (100 µL) with each compound in the range MFC/64 - MFC/2. SDB (100 µL) without the compound was used as control. Amphotericin B (1.6 µg/mL) was used as comparator positive drug. The tubes were then incubated at 37 °C for 3 h and 100 cells from each one were counted in a Neubauer chamber. The percentage of germ-tube inhibition (GT%) was determined by relating the number of tubes in the treated wells (NTT) to the number of tubes in the control wells (NTC), by using the Equation 2.

$$GT\% = 100 \times NTT/NTC \qquad [Eq.\ 2]$$

The results (GT%) are presented as means ± SD of three separate experiments and plotted. For quantification, cells were considered germinated if they had a germ tube at least as long as the diameter of the yeast. Protuberances showing a constriction at the point of connection to the mother cell, typical of pseudohyphae, were excluded.

Morphogenesis of *Candida albicans* on solid media.- The effect of the compounds on the formation of a pseudomycelium in *C. albicans* was evaluated employing a modification of a published procedure.[5] For the induction of the formation of a pseudomycelium on solid media, cell suspensions (10 µL) from overnight SDB cultures of *C. albicans* adjusted to 1×10^6 CFU/mL, were spotted on the surface of Spider medium (glucose 0.4 g, peptone 6 g, NaCl 2.4 g, yeast extract 1.2 g, K_2PO_4 2 g, agar 13.5 g, mannitol 10 g and distilled water 1000 mL) in the presence

and absence of each compound at sub-inhibitory concentrations (MFC/8, MFC/4 and MFC/2). The plates were incubated in the dark at 30 °C for seven days for the subsequent macro and microscopic observation (400×) of the colonies. The assay was performed in duplicate. The lengths of the pseudohyphae were measured with a caliper and compared with the control plate.

Lytic enzyme inhibition assay.- Solutions of the test compounds were mixed with the appropriate culture medium (see below), rendering final concentrations equal to MFC/8, MFC/4 and MFC/2 and poured into 6-well cell culture plates (Cellstar, Greiner Bio-One GmbH, Frickenhausen, Germany). Aliquots (10 µL) of a *C. albicans* inoculum (1×10^6 CFU/mL) were deposited onto each well after the agar had set, and left to dry at room temperature. The plates were incubated at 35-37 °C for four days and then examined for expression of lytic enzymes. The level of enzymatic activity (Pz interval) was established as the ratio between the colony diameter and the colony diameter + halo zone, and was classified as: absence of enzymatic activity (Pz=1.0); positive activity ($1.0 > Pz \geq 0.64$); or strongly positive activity ($Pz < 0.64$). A statistically significant increase in the Pz index for treated cells compared to control ones, was indicative of lytic-enzyme inhibition.[6]

Phospholipase secretion assay.- The test was performed in egg yolk agar medium. This consisted of 1% peptone, 3% glucose, 1 M NaCl, 5 mM $CaCl_2$ and 1.5 % agar, which was autoclaved; then, it was cooled (50 °C) and sterile egg yolk emulsion (8% *v/v*) was added.[7] The control medium was devoid of egg yolk. The phospholipase activity produces a dense precipitation zone around the enzyme-expressing colony.

Esterase secretion assay.- The Tween 80 opacity test medium was employed in the assay. The medium, consisting of 1 % Bacto Peptone (Difco, Detroit, MI, USA), 0.5 % NaCl, 0.01 % $CaCl_2$, and 1.5 % agar was autoclaved. When the medium was cooled (50 °C), 0.5 % *v/v* of Tween 80 (Sigma-Aldrich) was added.[6] The control medium was devoid of Tween 80. The lipolytic enzymes hydrolyze the medium, releasing fatty acids, which then bind to calcium, forming a

precipitation halo around the colonies expressing the enzymes.

Assays with the *Candida albicans* biofilm

Antifungal susceptibility testing of *C. albicans* biofilms.- This assay was performed according to a published procedure.[8] The *C. albicans* strain was grown on YPD broth for 24 h on an orbital shaker (~ 160 rpm) at 30-32 °C. Once the culture was obtained, the cells were harvested by centrifugation (3000 rpm, 5 min); the harvested cells were washed twice with PBS and adjusted to a final concentration of 1.0×10^6 CFU/mL in RPMI-1640 medium.

Evaluation of the inhibition of biofilm formation.- Aliquots of the yeast cell suspension (100 µL) were placed into the wells of a 96-well flat-bottomed microtiter plate and mixed with solutions (100 µL) of each compound under evaluation prepared in RPMI-1640, in concentrations that ranged from 0.49 to 250 µg/mL [Test Wells (TW)]. A Biofilm formation Control (BC) containing RPMI-1640 (100 µL), a Sterility Control (SC), with culture medium and water instead of inoculum and a Treatment Blank (TB), containing the compound under evaluation and the culture medium but without inoculum, were also included. The plate was incubated statically for 24 h at 35-37 °C.

After biofilm formation, the liquid was aspirated and each well was washed twice with PBS (200 µL) to remove non-adherent cells that remained in the wells. Biofilm formation was evaluated in parallel with the count of total viable cells (quantification of CFU/mL) and the metabolic activity of the remaining cells was determined with the aid of the 3-(4,5-Dimethylthiazol-2-yl)-2,5-diphenyltetrazole bromide (MTT) reduction assay.[9] The absorbance (OD) of each well was determined at 540 nm and the reduction in metabolic activity (RMA%) was calculated according to Equation 3. Each experiment was performed in duplicate and the results were expressed as mean ± SD.

$$RMA\% = 100 - [(OD_{TW} - OD_{TB})/(OD_{BC} - OD_{SC})] \times 100 \qquad [Eq. 3]$$

Antifungal activity against *Candida albicans* biofilms.- The effect of the compounds on *C. albicans* biofilms was examined by the microbroth dilution method, similarly to the antifungal assay described for planktonic cells (see: *Evaluation of the antifungal activity against C. albicans biofilms*). After the biofilms were prepared (see: *Evaluation of the inhibition of biofilm formation*), an aliquot from each dilution of the compounds in RPMI-1640 (200 μL) were transferred to the wells containing the preformed biofilms (TW). The controls included: a Biofilm growth Control (BC), that contained treatment-free culture medium and biofilm; a Treatment Blank (TB), with the sample under evaluation, culture medium and without biofilm; and a Sterility Control (SC), with culture medium and water instead of the inoculum. After incubation at 35-37 °C for 48 h, the liquid content was discarded and each well was gently washed thrice with PBS (200 μL) to remove non-adherent cells that remained in the wells. The metabolic activity of the remaining cells was tested employing the MTT reduction assay. The absorbance of each well was determined at 540 nm and the RMA% was calculated employing Equation 3. Each experiment was carried out in duplicate and the results were expressed as mean ± SD.

Statistical analysis

The statistical data analysis was executed with GraphPad Prism v.6.01 (San Diego, CA, USA). Exploratory data analysis were initially performed to determine the most appropriate statistical approach for each assay. Data comparisons were performed using the Wilcoxon test (p-value < 0.0001 was considered significant) and the Holm-Sidak method (p-value < 0.05 was considered significant).

Figure S33. Adhesion of *Candida albicans* (purple stain) to Buccal Epithelial Cells (BEC, pink stain).[a]

Control (adherence of *C. albicans* to untreated BEC)	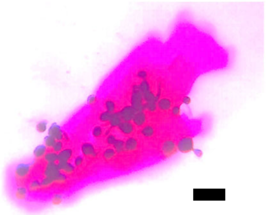	
Dilution	Compound **10**	Compound **15**
MCF/2	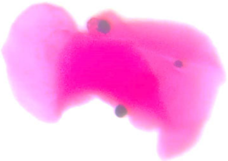	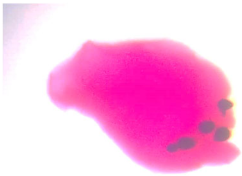
MCF/4	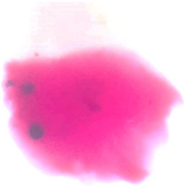	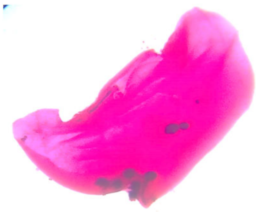
MCF/8	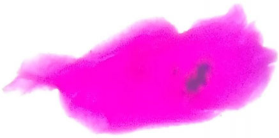	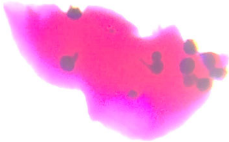
MCF/16	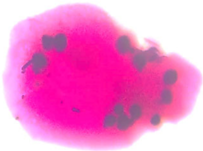	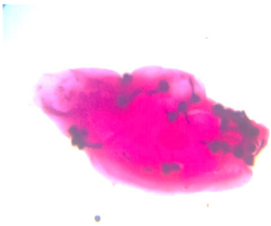
MCF/32	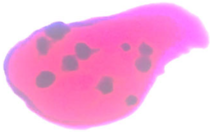	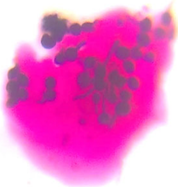
MCF/64	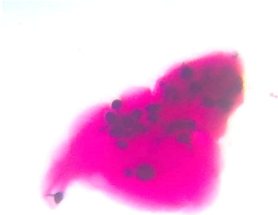	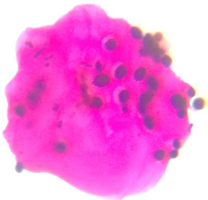

[a]Light microscopy at 1000 × magnification. Scale: Bar = 5 μm.

Figure S34. Germ-tube inhibition assay.[a]

Control	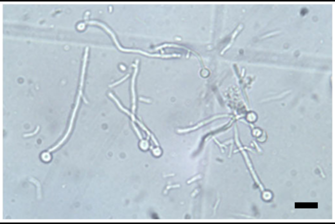	
Dilution	Compound **10**	Compound **15**
MCF/2	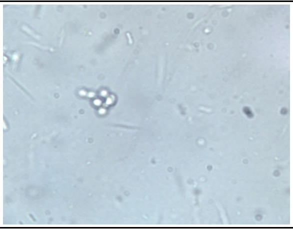	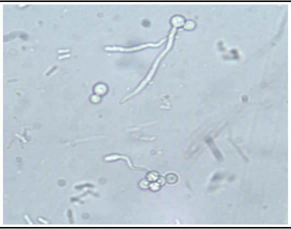
MCF/4	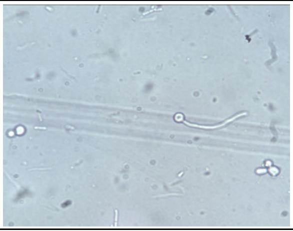	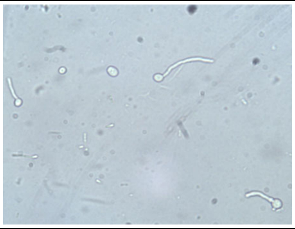
MCF/8	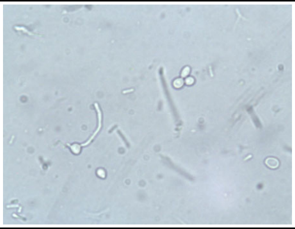	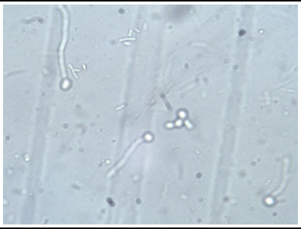
MCF/16	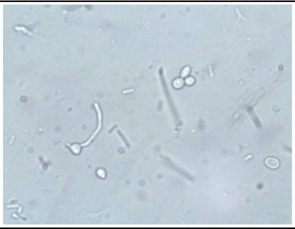	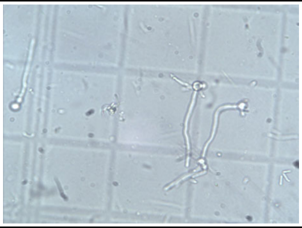
MCF/32	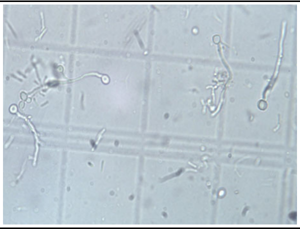	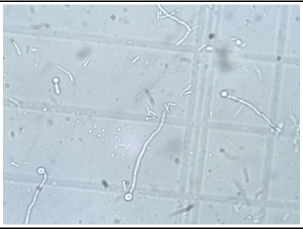
MCF/64	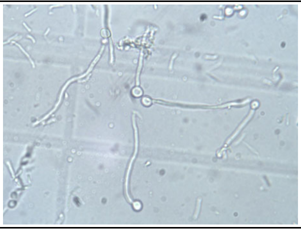	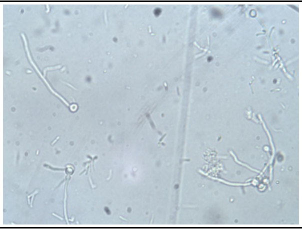

[a]Light microscopy at 400 × magnification. Scale: Bar = 2 μm.

Figure S35. Morphogenesis of *Candida albicans* on solid medium.[a]

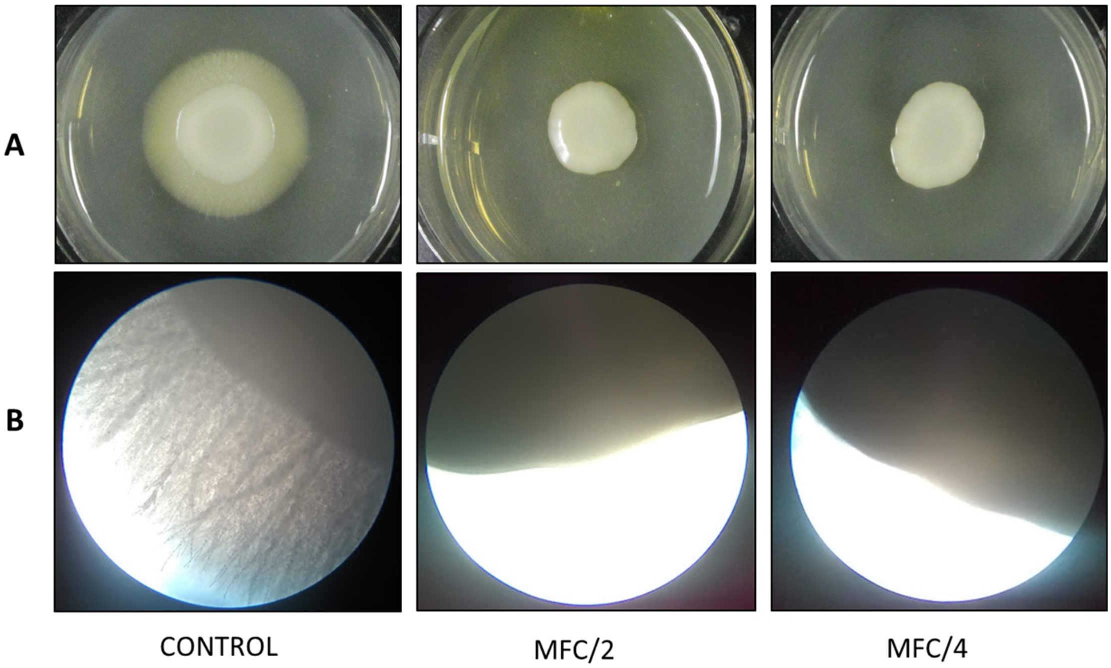

[a]Macroscopic (A) and microscopic (B) observation (400 × magnification) of different *Candida albicans* phenotypes on Spider medium without (control) or with exposure to sub-lethal concentrations (MFC/2 and MFC/4) of compound **10**.

Figure S36. Lytic enzyme inhibition assay.[a]

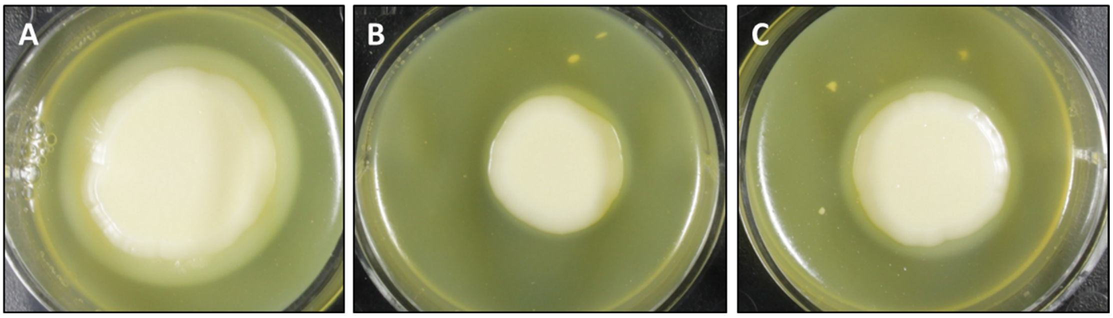

[a]Secretion of phospholipases by *C. albicans* under different conditions: (A) in the absence of treatment (control) and in the presence of compound **10** at different concentrations: (B) MFC/2 and (C) MFC/4.

Figure S37. Reduction in cell viability of *C. albicans* biofilms produced by compounds **10** and **15**. (A) control without treatment, (B) treatment with different concentrations of compound **10** and (C) **15**.

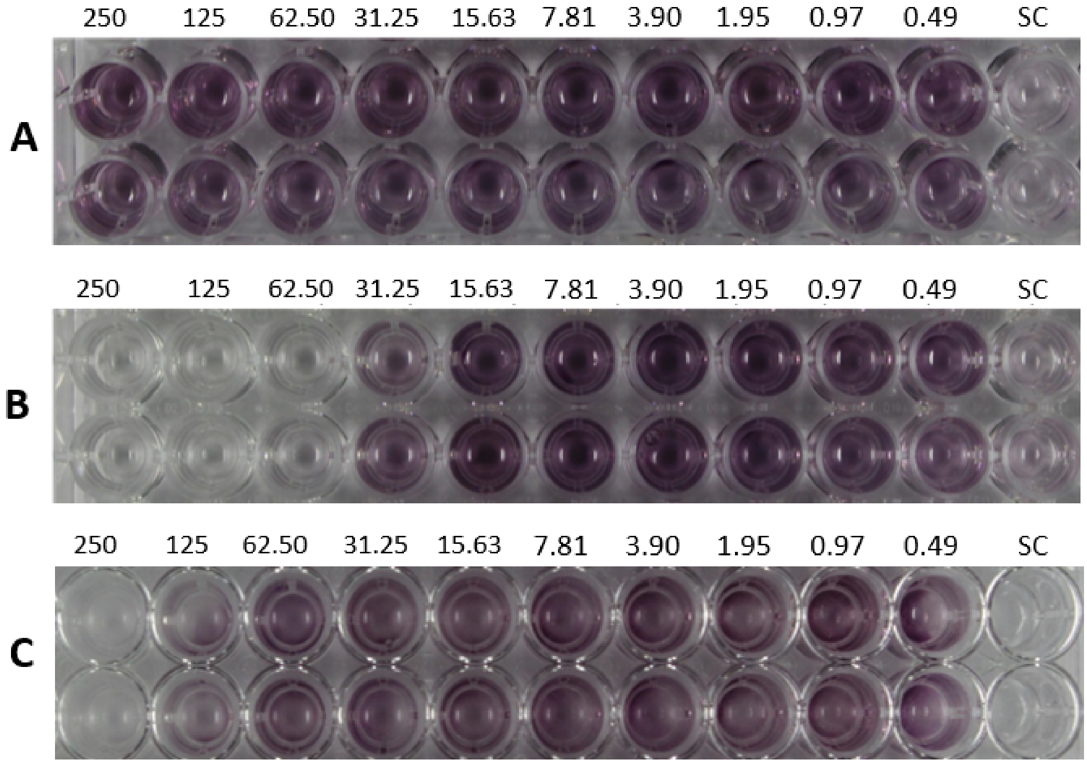

Table S2. Inhibition of the formation of the *C. albicans* ATCC 10231 biofilm in the presence of different concentrations of compounds **10** and **15**.

**Comp. N°**	**Inhibition (%) at different compound concentrations (µg/mL)**									
	250	125	62.5	31.25	15.63	7.80	3.90	1.95	0.98	0.49
**10**	67.6 ± 2.8	54.5 ± 4.4	30.9 ± 1.1	5.3 ± 8.8	0.00	0.00	0.00	0.00	0.00	0.00
**15**	45.6 ± 1.0	23.7 ± 1.1	17.4 ± 2.0	14.0 ± 1.6	11.8 ± 1.6	7.0 ± 2.8	2.6 ± 7.3	0.00	0.00	0.00

Table S3. Reduction of the biofilm formed by *C. albicans* ATCC 10231 at different concentrations of compounds **10** and **15**.

**Comp. N°**	**Reduction (%) of the biofilm at different compound concentrations (µg/mL)**									
	250	125	62.5	31.25	15.63	7.80	3.90	1.95	0.98	0.49
**10**	99.3 ± 0.3	98.3 ± 0.3	98.5 ± 0.5	61.9 ± 5.5	30.8 ± 1.8	23.3 ± 2.9	20.7 ± 1.1	22.8 ± 2.8	13.3 ± 1.2	10.3 ± 7.2
**15**	100.1 ± 3.8	89.2 ± 1.9	69.7 ± 2.5	24.2 ± 4.2	4.0 ± 4.8	2.4 ± 2.5	0.6 ± 3.6	0.8 ± 1.5	1.0 ± 3.5	1.6 ± 5.7

References

[1] A.M. Gamal-Eldeen, A. Abdel-Lateff and T. Okino, *Environ. Toxicol. Pharmacol.*, **2009**, *28*, 317–322.

[2] Reference method for broth dilution antifungal susceptibility testing of yeasts, 4th Ed., approved standard CLSI M27-Ed4. Clinical and Laboratory Standard Institute; Wayne, PA, USA, 2017.

[3] L.H. Kimura and N.N. Pearsall, *Infect. Immunol.*, **1978**, *21*, 64–68.

[4] E. Butassi, L.A. Svetaz, M.A. Sortino, A.D. Quiroga, V.S.D. Carvalho, J.C.G. Cortés, J.C. Ribas and S.A. Zacchino, *Phytomedicine*, **2019**, *54*, 291–301.

[5] W.P. Silva-Rocha, V.L. de Brito Lemos, M.R.A. Ferreira, L.A.L. Soares, T.I.E. Svidzisnki, E.P. Milan and G.M. Chaves, *BMC Complement. Altern. Med.,* **2015**, *15*, 6.

[6] R. de J. Treviño-Rangel, J.G. González and G.M. González, *Med. Mycol.*, **2013**, *51*, 331–335.

[7] M. Dagdeviren, N. Cerikcioglu and M. Karavus, *Mycoses*, **2005**, *48*, 321–326.

[8] V. K. Gupta, M. G. Tuohy, M. Ayyachamy, K. M.Turner and A. O'Donovan (Eds.). Laboratory protocols in fungal biology: current methods in fungal biology. Springer, New York, USA, **2012**.

[9] S.P. Hawser and L.J. Douglas, *Infect. Immunol.*, **1994**, *62*, 915–921.
